# Oncofetal MCB1 Is a Functional Biomarker for HCC Personalized Therapy

**DOI:** 10.1002/advs.202401228

**Published:** 2024-10-14

**Authors:** Daimin Xiang, Junyu Liu, Yichuan Wang, Dingtao Hu, Cheng Zhang, Tanlun Zeng, Weiqi Jiang, Xijun Liang, Wei Dong, Wen Sun, Li Xu, Hengyu Li, Yihai Shi, Jian Zhang, Hui Liu, Jin Ding

**Affiliations:** ^1^ Clinical Cancer Institute Center for Translational Medicine Naval Military Medical University Shanghai 200433 China; ^2^ Medical Innovation Center Shanghai East Hospital School of Medicine Tongji University Shanghai 200120 China; ^3^ Institute of Hepatobiliary and Pancreatic Surgery Department of Hepatobiliary and Pancreatic Surgery Shanghai East Hospital School of Medicine Tongji University Shanghai 200120 China; ^4^ National Center for Liver Cancer Naval Military Medical University Shanghai 200433 China; ^5^ Department of Pathology Third Affiliated Hospital of Naval Military Medical University Shanghai 200438 China; ^6^ Department of Liver Surgery Collaborative Innovation Center for Cancer Medicine Sun Yat‐sen University Cancer Center Guangzhou 510060 China; ^7^ Department of Breast and Thyroid Surgery Changhai Hospital Naval Military Medical University Shanghai 200433 China; ^8^ Department of Gastroenterology Shanghai Pudong New Area Gongli Hospital Shanghai 200135 China; ^9^ The State Key Laboratory of Cancer Biology Department of Biochemistry and Molecular Biology The Fourth Military Medical University Xi'an 710032 China; ^10^ Department of Hepatic Surgery Third Affiliated Hospital of Naval Military Medical University Shanghai 200438 China

**Keywords:** drug resistance, early diagnosis, hepatocellular carcinoma, MCB1, tumor‐initiating cells

## Abstract

Hepatocellular carcinoma (HCC) is one of the leading causes of cancer‐related death worldwide and lacks biomarkers for personalized therapy. Herein, it is reported that MCB1 could be a novel oncofetal protein that is upregulated in the preneoplastic lesions and serum of early HCC patients. Functional studies reveal that MCB1 modulated p53 protein degradation to promote T‐IC generation and drive HCC initiation. Furthermore, the MCB1/p53 axis is shown to determine the responses of hepatoma cells to conventional chemotherapeutics and predict transcatheter arterial chemoembolization (TACE) benefits in patients. Importantly, MCB1 can mediate sorafenib/lenvatinib resistance by downregulating two essential drug targets fibroblast growth factor receptor 1 (FGFR1) and vascular endothelial growth factor receptor 3 (VEGFR3) expression in a proteasome‐dependent manner. Patient‐derived tumor organoids (PDOs), patient‐derived xenografts (PDXs), and patient cohorts analysis suggested that MCB1 levels in HCCs may determine the distinct responses to conventional therapeutics and targeted drugs. Furthermore, treatment of targeted drugs‐resistant HCC with adeno‐associated virus (AAV) targeting MCB1 or a proteasome inhibitor restores targeted drug response, suggesting their clinical significance in HCC combinational therapy. In conclusion, these findings demonstrate that MCB1 could act as a driver for HCC initiation, a contributor to drug resistance, and a biomarker for individualized HCC therapy.

## Graphical abstract

Oncofetal protein MCB1 promotes T‐IC generation and drives HCC initiation through accelerating p53 degradation, and MCB1/p53 axis determines the responses of HCC cells to conventional chemotherapeutics. MCB1 also mediates sorafenib/lenvatinib resistance by downregulating FGFR1 and VEGFR3 expression in a proteasome‐dependent manner and serves as a potential biomarker for individualized HCC therapy.

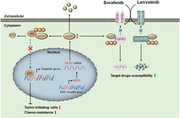

## 1 Introduction

Hepatocellular carcinoma (HCC) is one of the most common malignancies, and its incidence has been rising worldwide.^[^ ^1^ ^]^ Due to the inconspicuous symptoms of early HCC, most patients are diagnosed at an advanced stage and thus miss the best time for surgical treatment. Approximately 80% of patients with advanced HCC develop postoperative recurrence.^[^ ^2^ ^]^ Transcatheter arterial chemoembolization (TACE) and tyrosine kinase inhibitors (TKIs), such as sorafenib and lenvatinib, are primary therapeutic options for HCC patients with unresectable tumors.^[^ ^3^, ^4^, ^5^ ^]^ Due to the high heterogeneity of HCC, only a few patients respond well to TACE or TKIs. Most patients are resistant to these therapeutics and suffer from adverse effects to varying degrees. The lack of reliable biomarkers and the selection of patients who will benefit from these therapeutics remain major challenges to date. Therefore, the identification of applicable biomarkers that can predict drug responses in HCC patients for individualized therapy is urgently needed.

The ubiquitin‐proteasome system (UPS) plays a vital role in cellular protein degradation and maintenance of cell homeostasis.^[^ ^6^ ^]^ An abnormal UPS may cause serious diseases, including various types of cancer.^[^ ^7^ ^]^ It is generally accepted that identifying the key proteins involved in the protein degradation process in cancer cells is of great importance for elucidating the mechanism underlying carcinogenesis and developing novel diagnostic biomarkers and therapeutic targets. Ubiquitination is an important post‐transcriptional modification that affects the localization, stability, and activity of the target protein.^[^ ^8^ ^]^ Substrate recognition by the proteasome is presumed to be mediated by one or more receptor(s) with affinity for multiubiquitin chains. Multiubiquitin chain‐binding protein (MCB1) is a ubiquitin receptor subunit of the 26S proteasome.^[^ ^9^ ^]^ This molecule has ubiquitin‐interacting motifs (UIMs) that recognize polyubiquitin chains and mediate the degradation of ubiquitinated proteins.^[^ ^10^ ^]^ Nevertheless, the role of MCB1 in human disease remains unclear.

In this study, we discovered that MCB1 drove HCC initiation and chemoresistance by downregulating the expression of p53 and TKI targets, which suggests that MCB1 could be a novel biomarker for early HCC diagnosis and individualized therapy.

## 2 Results

### 2.1 Expression of MCB1 Is Elevated in Early HCC and Correlates with Drug Resistance

**Figure 1:**
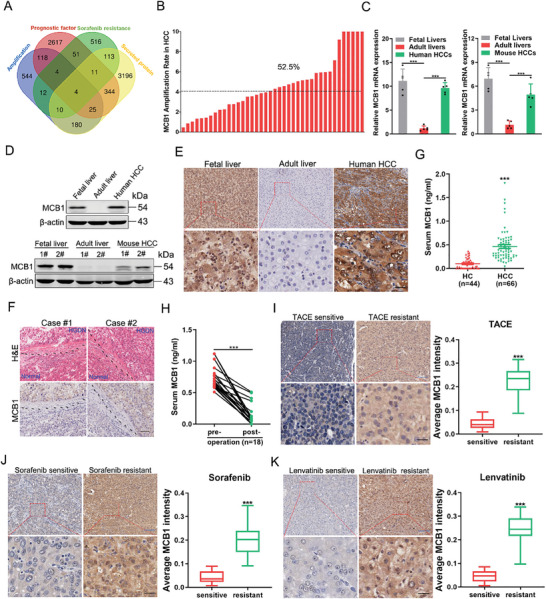
Oncofetal protein MCB1 levels are elevated in early HCC and correlate with drug resistance. A) Venn diagrams showing the amplified genes and prognostic factors in TCGA data, sorafenib‐resistant genes in literature, and secreted proteins. The GISTIC algorithm was used to perform a CNV analysis on the TCGA liver cancer cohort and those genes exhibiting a CNV value >0.3 were considered as amplificated genes. Liver cancer patients in the TCGA cohort were divided into high and low‐expression groups based on the medium expression value of each gene. The prognostic genes were selected if the survival comparison between the two groups yielded a p‐value <0.05. The R package “limma” was used to obtain sorafenib resistance‐related genes from GSE109211. The threshold setting of the DEGs was |logFC|>1.5 and P. adj < 0.05. B) MCB1 gene amplification rate in HCC was determined by real‐time PCR analysis (*n* = 40). C) The mRNA expression of MCB1 in human or murine fetal livers (*n* = 5), adult livers (*n* = 5), and HCC tissues (*n* = 5) was detected by real‐time PCR. Mouse HCC tissues were collected from the DEN‐induced mouse hepatocarcinogenesis model. Data are presented as mean ± SD. D) The protein expression of MCB1 in fetal livers, adult livers, and HCC tissues from humans or mice was examined by western blotting. E) Representative views of IHC staining of MCB1 in human fetal livers, adult livers, and HCC tissues. Blue scale bar, 5 µm. Black scale bar, 25 µm. F) H&E staining and IHC for MCB1 in human HGDN and paired adjacent normal liver tissues. Scale bar, 50 µm. G) ELISA analysis of MCB1 in the serum of healthy controls (HCs) (*n* = 44) and early HCC patients (*n* = 66). H) ELISA assay showed the decrease of serum MCB1 in 18 of 23 HCC patients after tumor resection. I–K) MCB1 expression was compared in TACE‐sensitive (*n* = 30) and TACE‐resistant (*n* = 30) HCC patients; sorafenib‐sensitive (*n* = 30) and sorafenib‐resistant (*n* = 30) HCC patients; lenvatinib‐sensitive (*n* = 30) and lenvatinib‐resistant (*n* = 30) HCC patients. Blue scale bar, 5 µm. Black scale bar, 25 µm. Data are presented as mean ± SD. Unless otherwise indicated, *p*‐values were determined by unpaired student's *t* test (two‐tail) and ^***^, which indicate *p*‐value < 0.001.

To identify novel diagnostic and therapeutic biomarkers in HCC, we obtained amplified genes (897) and prognostic genes (3164) from The Cancer Genome Atlas (TCGA), sorafenib‐resistant genes (721) from the literature^[^ ^11^ ^]^ as well as secretary proteins (3883) from The Human Protein Atlas (HPA) and the literature^[^ ^12^ ^]^ (**Figure** **1**A; Table S1, Supporting Information). In total, four genes were identified from all four groups, among which MCB1 was the top amplified gene in HCC (Figure 1B). Strikingly, MCB1 transcript and protein levels were relatively high in fetal livers but were low in adult livers (Figure 1C–E). Expression of MCB1 was reactivated in HCCs from both mice and humans ((Figure 1C–E), suggesting that MCB1 could be a novel oncofetal protein. It is widely accepted that most HCCs develop from high‐grade dysplastic nodules (HGDNs), which are precancerous lesions of tumorigenesis.^[^ ^13^ ^]^ IHC results showed that MCB1 levels were significantly higher in HGDNs, suggesting that MCB1 might play a role in hepatocyte transformation (Figure 1F). As expected, MCB1 transcript and protein levels were notably increased in HCC tissues compared with adjacent noncancerous liver tissues (Figure S1A–C, Supporting Information). High MCB1 levels were associated with aggressive features of HCC and poor survival of patients (Figure S1D–F; Table S2, Supporting Information). In addition, the serum MCB1 levels were increased in early HCC patients compared with healthy donors (Figure 1G). Moreover, the serum MCB1 levels decreased after tumor resection (Figure 1H), implying that serum MCB1 could be produced by tumor cells. Notably, MCB1 expression was higher in HCC tissues resistant to TACE, sorafenib, or lenvatinib therapy than in those sensitive to the treatments (Figure 1I–K), suggesting that MCB1 expression is closely associated with HCC chemoresistance.

### 2.2 MCB1 Facilitates Hepatocyte Transformation and HCC Initiation

To investigate the oncogenic role of MCB1 in the liver, we introduced MCB1 into the normal hepatocyte cell lines HL7702 and THLE3 (Figure S2A, Supporting Information). Interestingly, ectopic MCB1 expression enhanced the expression of tumor‐initiating cell (T‐IC) markers and promoted the generation of T‐ICs (Figure S2B,C, Supporting Information). Moreover, ectopic MCB1 induced colony formation in vitro (Figure S2D, Supporting Information) and tumor formation in vivo (Figure S2E, Supporting Information). The xenografted tumors exhibited the HCC phenotype with strong alpha fetal protein (AFP) staining (Figure S2F, Supporting Information).

**Figure 2:**
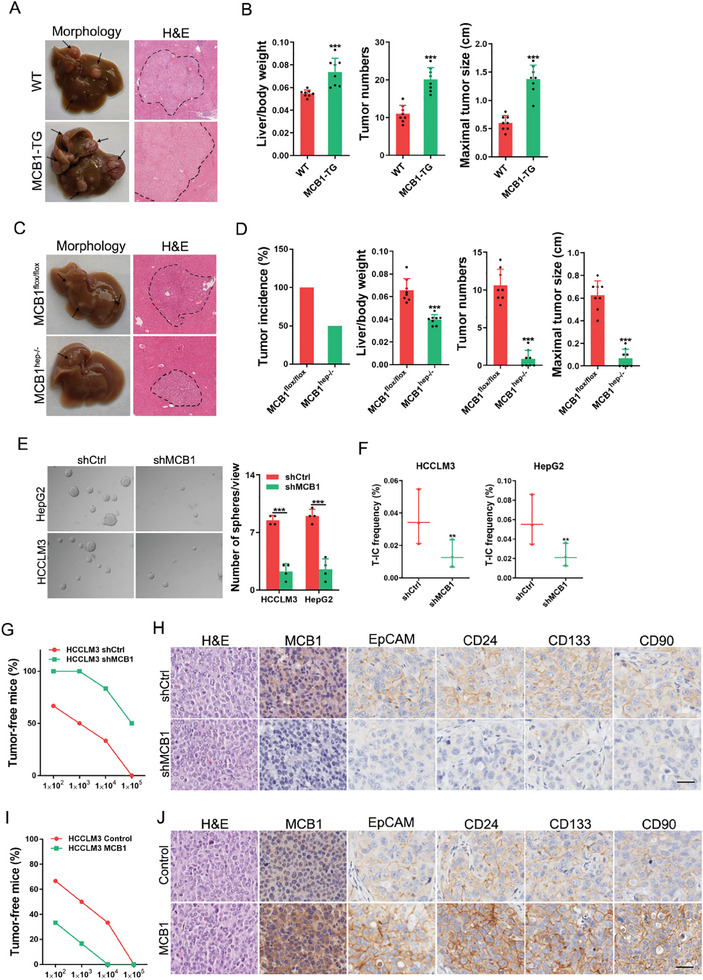
MCB1 drives liver oncogenesis by promoting T‐IC generation. A) Representative images and H&E staining of the MCB1‐TG and WT male mice at 5 months after DEN injection. B) Liver/body weight, tumor number, and max tumor size in mouse liver tissues in the MCB1‐TG and WT mice (*n* = 8). Data are presented as mean ± SD. C) Representative images and H&E staining of the MCB1^hep‐/−^ and MCB1^flox/flox^ mice at 5 months after DEN injection. D) Tumor incidence, liver/body weight, tumor number, and max tumor size in mouse liver tissues in the MCB1^hep‐/−^ and MCB1^flox/flox^ mice (*n* = 8). Data are presented as mean ± SD. E) Representative images of hepatoma spheroids generated from the MCB1 knockdown and control hepatoma cells. The number of spheroids was counted and compared (*n* = 4). Data are presented as mean ± SD. F) The frequency of liver T‐ICs in the MCB1 knockdown and control hepatoma cells was compared by an in vitro limiting dilution assay. G) MCB1 knockdown and control hepatoma cells were inoculated into NOD‐SCID mice subcutaneously (*n* = 6), and tumorigenicity was evaluated two months post‐inoculation. H) Representative images of immunohistochemical staining of liver T‐IC markers in the MCB1 knockdown and control hepatoma cells that formed xenografted tumors. Scale bar, 25 µm. I) MCB1‐overexpressing and control hepatoma cells were subcutaneously inoculated into NOD‐SCID mice (*n* = 6), and tumorigenicity was evaluated two months post‐inoculation. J) Representative images of immunohistochemical staining of liver T‐IC markers in MCB1 overexpression and control hepatoma cells formed xenografted tumors. Scale bar, 25 µm. Unless otherwise indicated, p‐values were determined by unpaired student's *t*‐test (two‐tail) and ^**^, ^***^, indicate *p*‐value < 0.01, < 0.001, respectively.

To further explore the role of MCB1 in hepatocarcinogenesis, we generated MCB1 transgenic mice (MCB1‐TG) and constructed hepatocyte‐specific MCB1 knockout mice (MCB1^hep‐/−^) (Figure S2G–J, Supporting Information). Post the administration of diethylnitrosamine (DEN), MCB1‐TG, or MCB1^hep‐/−^ mice were intraperitoneally injected with CCl_4_ twice a week for two months (Figure S2K, Supporting Information). Hepatic tumor foci were observed in all mice in the control group and the MCB1‐TG group (**Figure** **2**A). The tumor numbers, maximal tumor sizes, and liver‐to‐body weight ratios were significantly increased upon MCB1 overexpression in hepatocyte (Figure 2B). Consistently, reduced HCC formation was observed in the MCB1^hep‐/−^ mice (Figure 2C). Hepatocyte‐specific deletion of MCB1 drastically reduced the tumor incidence, numbers, maximal tumor sizes, and liver‐to‐body weight ratios (Figure 2D). Ki67 and caspase‐3 staining showed increased hepatoma cell proliferation and decreased cell apoptosis in the MCB1‐TG mice (Figure S2L, Supporting Information). Consistently, a decrease in hepatoma cell proliferation and an increase in hepatoma cell apoptosis were observed in the MCB1^hep‐/−^ mice (Figure S2M, Supporting Information). No significant difference in levels of aspartate aminotransferase (AST) and alanine aminotransferase (ALT) was found in MCB1‐TG or MCB1^hep‐/‐^ group and the control groups (Figure S2N,O, Supporting Information), suggesting that MCB1 overexpression or deletion had marginal influence on liver injury triggered by DEN stimulation. Additionally, no difference in hepatic macrophage infiltration was observed between the MCB1‐TG or MCB1^hep‐/‐^ mice and their control mice (Figure S2P,Q, Supporting Information). Interestingly, the expression of T‐IC‐related markers such as EpCAM, CD24 and CD133 was significantly increased in MCB1‐TG mice and decreased in MCB1^hep‐/−^ mice (Figure S2L,M, Supporting Information), suggesting that MCB1 gives rise to T‐ICs and drives HCC initiation. Consistently, the RNA sequencing data from HCC tissues of animal models showed that the stemness‐associated genes were notably increased in MCB1‐TG mice and decreased in MCB1^hep‐/−^ mice, when compared with WT mice (Figure S2R,S, Supporting Information).

### 2.3 MCB1 Promotes the Expansion of Liver T‐ICs

Primary patient HCC cells and HCC cell lines were used to enrich T‐ICs by flow cytometric sorting or sphere formation assay. We found that MCB1 expression was increased in EpCAM^+^ and CD24^+^ primary HCC cells (Figure S3A, supporting information). Furthermore, MCB1 expression was higher in spheroids than in attached cells (Figure S3B, Supporting Information), and the difference could be restored to some extent during reattachment, with the cells undergoing differentiation (Figure S3C, Supporting Information). Consistent results were achieved in HCC cell lines (Figure S3D–G, Supporting Information). As shown in Figure S3H, supporting information, TCGA‐LIHC data analysis revealed that high MCB1 levels correlated with high tumor stemness scores.^[^ ^14^ ^]^

To explore the potential role of MCB1 in liver T‐ICs, we used MCB1 knockdown and overexpression HCC cell lines (Figure S3I,J, Supporting Information). Notably, the spheroid forming ability was impaired in the MCB1 knockdown HCC cells (Figure 2E) and was enhanced in the MCB1‐overexpressing HCC cells (Figure S3K, Supporting Information). Importantly, the T‐IC proportion was dramatically decreased in MCB1‐knockdown HCC cells (Figure 2F) and enhanced in the MCB1‐overexpressing HCC cells (Figure S3L, Supporting Information) in in vitro limiting dilution assay. More importantly, the tumor incidence was drastically reduced upon MCB1 interference in in vivo limiting dilution assay (Figure 2G). Fewer EpCAM^+^, CD133^+^, CD24^+^, or CD90^+^ T‐ICs were detected in the MCB1 knockdown cell‐formed xenografts compared with the control xenografts (Figure 2H). In contrast, MCB1 overexpression markedly enhanced tumor initiation in vivo (Figure 2I). Increased EpCAM^+^, CD133^+^, CD24^+^, or CD90^+^ liver T‐ICs were detected in the MCB1‐overexpressing cell‐formed xenografts compared with the control xenografts (Figure 2J). Conclusively, these results suggest that MCB1 may play a pivotal role in the expansion of liver T‐ICs.

### 2.4 P53 Is Required for MCB1‐Mediated HCC Initiation

**Figure 3:**
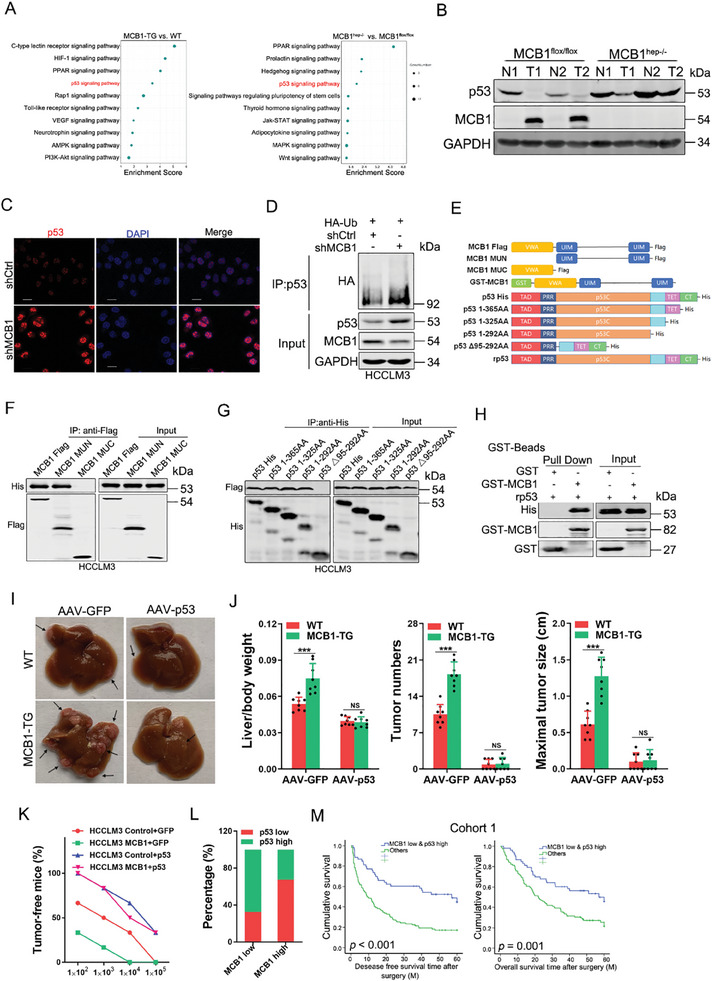
p53 is responsible for MCB1‐mediated liver cancer initiation. A) KEGG pathway enrichment of the top 10 signaling pathways according to those up‐regulated genes upon MCB1 overexpression or down‐regulated genes by MCB1 deletion compared with their WT control mouse livers at 5 months after DEN injection. B) Western blot analysis of tumors and peritumoral normal tissues in the livers from the MCB1^hep‐/−^ and MCB1^flox/flox^ mice at 5 months after DEN injection. C) Representative images of immunofluorescence staining of p53 in the MCB1 knockdown or control hepatoma cells. The nuclei were counterstained with 4′,6‐diamidino‐2‐phenylindole. Scale bar, 20 µm. D) MCB1 knockdown and control hepatoma cells were transfected with hemagglutinin (HA)‐tagged ubiquitin, and then, total ubiquitinated proteins were detected by western blotting for HA. E) Schematic illustration of p53 and MCB1 constructs. F) MCB1 truncations and His‐tagged P53 plasmids were transfected into HCCLM3 cells and then subjected to immunoprecipitation with agarose conjugated anti‐Flag antibody, and analyzed by immunoblotting with antibody against His and Flag. G) p53 truncations and Flag‐tagged MCB1 plasmids were transfected into HCCLM3 cells and then subjected to immunoprecipitation with agarose conjugated anti‐His antibody, and analyzed by immunoblotting with antibody against His and Flag. H) Direct binding of rp53 to rMCB1 using a GST pull‐down assay. I) Representative images of mouse livers at 5 months after DEN injection. J) Liver/body weight, tumor number, and max tumor size in mouse liver tissues in the WT + AAV‐GFP, WT + AAV‐p53, MCB1‐TG + AAV‐GFP and MCB1‐TG + AAV‐p53 groups (*n* = 8 male mice). Data are presented as mean ± SD. K) MCB1‐overexpressing cells and control hepatoma cells were infected with p53 overexpression virus or control virus and then subjected to an in vivo limiting dilution assay (*n* = 6). L) The correlation between MCB1 levels and p53 expression in HCC tissues from cohort 1 was evaluated using a chi‐square test. M) OS and DFS of the patients exhibiting low MCB1 and high p53 levels were compared with the other patients in cohort 1 using Kaplan‐Meier analysis. Unless otherwise indicated, *p*‐values were determined by unpaired student's *t*‐test (two‐tail) and ^***^, NS indicates *p*‐value < 0.001, not significant, respectively.

To explore the molecular mechanism underlying MCB1‐triggered hepatocarcinogenesis, we profiled gene expression in HCC from the MCB1‐TG or MCB1^hep‐/‐^ and WT mice using RNA sequencing. Kyoto Encyclopedia of Genes and Genomes (KEGG) enrichment analysis revealed that the p53 and PPAR signaling pathways were enriched from those up‐regulated genes upon MCB1 overexpression or down‐regulated genes by MCB1 deletion (**Figure** **3**A). Moreover, Gene Set Enrichment Analysis (GSEA) revealed that MCB1 overexpression or deletion resulted in the enrichment of gene sets related to the p53 pathway (Figure S4A,B, Supporting Information). Considering the essential role of p53 in tumorigenesis, we focused on the regulation of p53 by MCB1. Western blot assays revealed the upregulation of MCB1 expression in parallel with the reduction of p53 expression in murine orthotopic HCC cells, and the reduction was impaired upon MCB1 deficiency (Figure 3B). MCB1 interference increased p53 protein expression, and ectopic MCB1 expression decreased p53 protein levels in HCC cells (Figure 3C; Figure S4C,D, Supporting Information). Moreover, we found that MCB1 downregulated p53 expression through proteasome‐dependent degradation in HCC cells (Figure 3D; Figure S4E,F, Supporting Information). Furthermore, we delineated that UIMs of MCB1 directly binds to the central DNA‐binding domain of p53 (Figure 3E–H; Figure S4G,H, Supporting Information).

We further explored whether p53 is required for MCB1‐mediated HCC development. As shown in Figure S5A–D, supporting information, the self‐renewal, colony formation, and tumorigenesis triggered by ectopic MCB1 expression in hepatocytes was abrogated by p53 rescue. To confirm the role of p53 in MCB1‐promoted hepatocarcinogenesis, we injected adeno‐associated virus (AAV) specifically expressing murine p53 in hepatocytes (AAV/DJ‐ALBp‐GFP‐2A‐mp53, hereafter referred to as AAV‐p53) or its control (AAV/DJ‐ALBp‐GFP, hereafter referred to as AAV‐GFP) into the tail vein of DEN‐treated 4‐week‐old MCB1‐TG and control mice (Figure S5E, Supporting Information). Hepatocyte‐specific p53 expression in AAV‐p53‐infected mouse livers (5 months) was confirmed by immunoblotting (Figure S5F, Supporting Information). Macroscopic examination of livers revealed fewer and smaller tumors in the AAV‐p53‐infected MCB1‐TG and control mice (Figure 3I). Notably, p53 rescue not only reduced the tumor numbers, maximal tumor sizes, and liver‐to‐body weight ratios in the WT mice but also completely blocked MCB1‐enhanced HCC development in the MCB1‐TG mice (Figure 3J). In addition, p53 rescue abrogated the increased T‐IC markers as well as Ki67 and the decreased hepatoma cell apoptosis mediated by transgenic MCB1 expression (Figure S5G,H, Supporting Information). To further confirm whether p53 was involved in MCB1‐promoted liver T‐IC expansion, we designed a specific p53 siRNA. As expected, interference of p53 blocked the reduction in self‐renewal and liver T‐IC frequency in the MCB1 knockdown HCC cells (Figure S5I–K, Supporting Information). In addition, p53 overexpression abrogated the MCB1‐enhanced self‐renewal, liver T‐IC frequency and tumorigenicity of hepatoma cells (Figure S5L–N, Supporting Information, and Figure 3K). Consistently, TCGA‐LIHC data analysis showed that HCCs in MCB1^high^p53^low^ group exhibited higher stemness scores than those in MCB1^low^p53^high^ group (Figure S5O, Supporting Information). Pathway analysis was then performed and a series of cancer stemness‐related pathways were enriched, including Wnt signaling pathway, TGF‐β signaling pathway, Notch signaling pathway and Hedgehog signaling pathway etc. (Figure S5P, Supporting Information). Taken together, these data suggested that p53 is involved in MCB1‐promoted T‐IC expansion and HCC initiation.

We then explored the association between MCB1 expression and p53 levels in patient HCCs. A correlation between high MCB1 expression and low p53 levels was observed in patient HCC tissues (Figure 3L). Although low MCB1 (cohort 1, Figure S1F, Supporting Information) or high p53 (cohort 1, Figure S5Q, Supporting Information) in HCC predicts a good prognosis, HCC patients with both reduced MCB1 levels and elevated p53 expression displayed an even better prognosis (cohort 1, Figure 3M), suggesting an improved prognostic value of the combination of MCB1 and p53. In addition, TCGA‐LIHC data analysis also showed that HCC patients in the MCB1^high^ p53^low^ group exhibited shorter survival time compared to those in the MCB1^low^ p53^high^ group (Figure S5R, Supporting Information).

### 2.5 MCB1 May Serve as a Biomarker for HCC Personalized Chemotherapy

**Figure 4:**
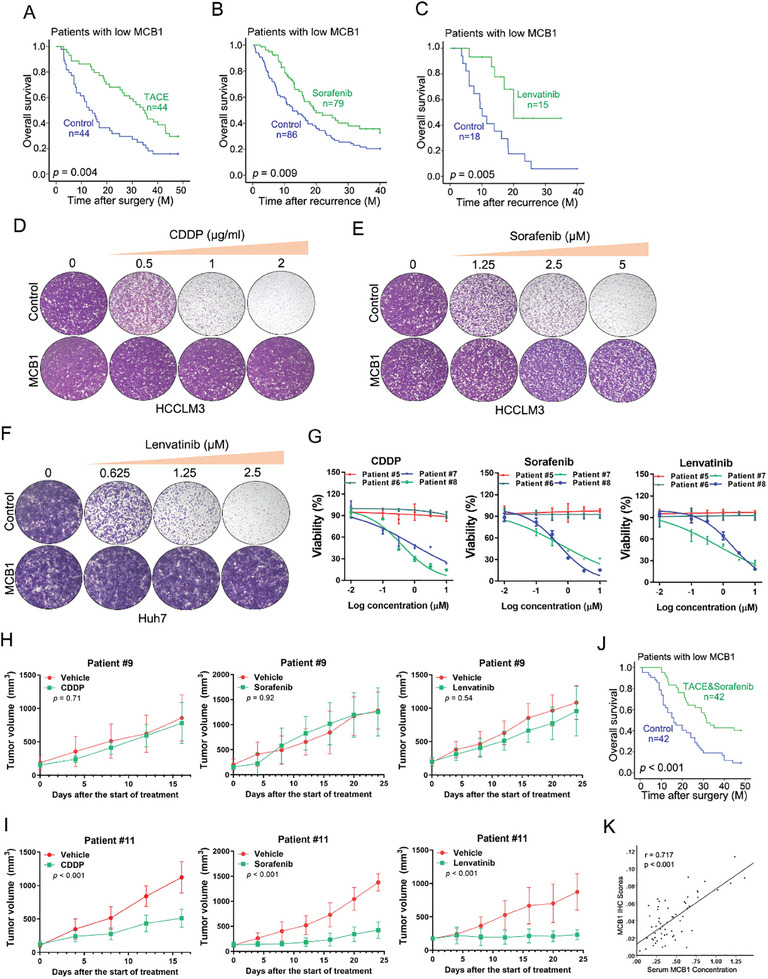
Significance of MCB1 in HCC personalized therapy. A) Overall survival rates of HCC patients with low MCB1 levels treated with TACE (*n* = 44) or not (*n* = 44) after surgery were compared using Kaplan‐Meier analysis (*p* = 0.004). B) Overall survival rates of HCC patients with low MCB1 levels treated with sorafenib (*n* = 79) or not (*n* = 86) after HCC relapse were compared using Kaplan‐Meier analysis (*p* = 0.009). C) Overall survival rates of HCC patients with low MCB1 levels treated with lenvatinib (*n* = 15) or not (*n* = 18) after HCC relapse were compared using Kaplan‐Meier analysis (*p* = 0.005). D) HCCLM3 MCB1 and control cells were treated with CDDP for 10 days and their colony growth was examined. E) HCCLM3 MCB1 and control cells were treated with sorafenib for 10 days and their colony growth was examined. F) Huh7 MCB1 and control cells were treated with lenvatinib for 10 days and their colony growth was examined. G) PDOs derived from primary HCCs with high MCB1 levels (Patients #5‐6) or low MCB1 levels (Patients #7‐8) were treated with CDDP, or targeted drugs for 7 days, and their cell survival curves were calculated. Data are presented as mean ± SD. H, I) PDXs derived from primary HCCs with high MCB1 levels (Patients #9) or low MCB1 levels (Patients #11) were treated with CDDP (2 g/kg body weight), sorafenib (30 mg/kg body weight), lenvatinib (4 mg/kg body weight) or vehicle (*n* = 5 for each group). Xenograft growth was monitored. Data are presented as mean ± SD. J) Overall survival rates of HCC patients with low MCB1 levels received postsurgical TACE and sorafenib (*n* = 42) or not (*n* = 42) were compared using Kaplan‐Meier analysis (*p* < 0.001). K) Pearson correlation analysis between MCB1 levels in HCC tissues and those in the serum of HCC patients (*n* = 60). Unless otherwise indicated, *p*‐values were determined by unpaired student's *t*‐test (two‐tail).

MCB1 is located on human chromosome 1q21, and copy number amplification of this region correlates with chemoresistance in cancer,^[^ ^15^ ^]^ implying that MCB1 might be associated with chemoresistance in HCC. We initially studied the influence of MCB1 expression on the response of HCC to conventional chemotherapeutics and the targeted drugs in patients. Our results showed that HCC patients with low MCB1 expression exhibited superior prognosis following postoperative adjuvant TACE treatment, while patients with high MCB1 levels had poor response (**Figure** **4**A; Figure S6A, Supporting Information). Moreover, the patients with low MCB1 expression displayed longer survival time following sorafenib treatment after HCC relapse, while patients with high MCB1 levels exhibited no response to sorafenib therapy (Figure 4B; Figure S6B, Supporting Information). Among the patients received lenvatinib after HCC relapse, MCB1 low patients exhibited prolonged overall survival but MCB1 high patients did not (Figure 4C; Figure S6C, Supporting Information). Moreover, the MCB1‐overexpressing hepatoma cells were resistant to CDDP, sorafenib or lenvatinib in comparison with the control cells, which was demonstrated by a higher inhibitive concentration (Figure S6D, Supporting Information). Consistently, MCB1 overexpression impaired the growth inhibition and cell apoptosis induced by CDDP or targeted drugs in hepatoma cells (Figure 4D–F; Figure S6E–L, Supporting Information).

Next, the correlation between MCB1 levels and drug response was further explored using patient‐derived tumor organoids (PDOs) and patient‐derived xenografts (PDXs) (Figure S6M, Supporting Information). It was observed that PDOs from tumors with high MCB1 expression showed resistance to CDDP or targeted drug treatment (Figure 4G). Similarly, the growth of PDXs derived from primary HCCs with low MCB1 expression was dramatically suppressed upon CDDP or targeted drug treatment (Figure 4H and I and Figure S6N,O, Supporting Information). Moreover, the expression of Ki67, which is a classic proliferating cell marker, was decreased in CDDP or targeted drugs‐treated PDXs derived from tumors with low MCB1 levels (Figure S6P, Supporting Information). More importantly, Kaplan‐Meier analysis showed that the patients with low MCB1 levels in their primary HCCs had prolonged OS upon postsurgical TACE and sorafenib treatment, while patients with high MCB1 levels exhibited poor response (Figure 4J; Figure S6Q, Supporting Information). Collectively, these data suggest that MCB1 levels in HCC correlate with the therapeutic effect of CDDP or targeted drugs. In addition, Pearson correlation analysis revealed that MCB1 levels in primary HCC tissues were correlated with serum MCB1 levels (Figure 4K), suggesting that serum MCB1 might serve as a biomarker for HCC personalized chemotherapy.

### 2.6 MCB1 Promotes CDDP Resistance by Downregulating p53

Existing evidence showed that p53 downregulation or loss function was closely associated with CDDP resistance in numerous human cancers.^[^ ^16^, ^17^ ^]^ Consistent with previous studies, our data showed that knockdown of p53 decreased the susceptibility of hepatoma cells to CDDP‐induced apoptosis (Figure S7A,B, Supporting Information). Importantly, interference of p53 abrogated the MCB1 knockdown‐mediated decrease in cell viability and increase in cell apoptosis upon CDDP treatment (Figure S7C,D, Supporting Information). Moreover, introduction of p53 resensitize MCB1 overexpression hepatoma cells to CDDP treatment (Figure S7E,F, Supporting Information), which further suggesting that p53 was responsible for MCB1 mediated conventional chemoresistance.

### 2.7 MCB1 Downregulates FGFR1 and VEGFR3 Expression via Proteasome‐Dependent Degradation

**Figure 5:**
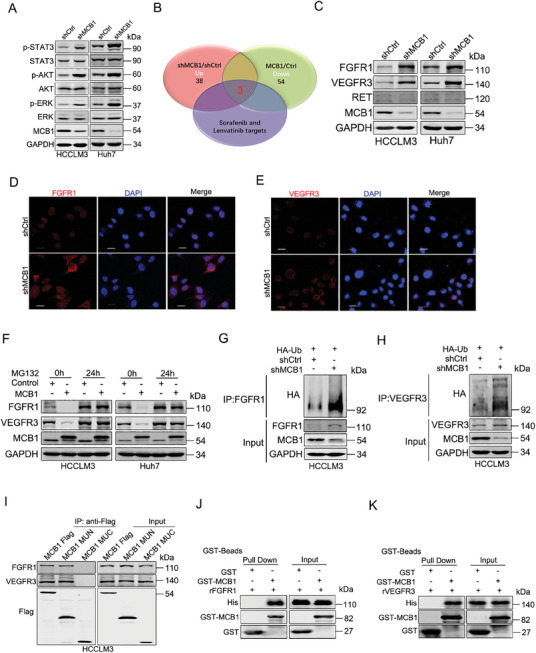
Direct interactions of FGFR1 or VEGFR3 and MCB1. A) Western blot analysis of the phosphorylation of STAT3, AKT, and ERK in the MCB1 knockdown cells and control hepatoma cells. B) Venn diagrams showing the genes with upregulated expression in the MCB1 knockdown HCC cells and the genes with downregulated expression in the MCB1‐overexpressing HCC cells and sorafenib and lenvatinib targets. C) Western blot analysis of the levels of FGFR1, VEGFR3, and RET in the MCB1 knockdown cells and control hepatoma cells. D) Representative images of immunofluorescence staining of FGFR1 in MCB1 knockdown or control hepatoma cells. The nuclei were counterstained with 4′,6‐diamidino‐2‐phenylindole. Scale bar, 20 µm. E) Representative images of immunofluorescence staining of VEGFR3 in MCB1 knockdown or control hepatoma cells. The nuclei were counterstained with 4′,6‐diamidino‐2‐phenylindole. Scale bar, 20 µm. F) MCB1 overexpression cells or control hepatoma cells were treated with MG132 (20 µm) for the indicated times and then subjected to western blot analysis. G, H) MCB1 knockdown cells and control hepatoma cells were transfected with hemagglutinin (HA)‐tagged ubiquitin, and then, ubiquitinated proteins were detected by western blotting for HA. I) MCB1 truncation plasmids were transfected into HCCLM3 cells and then subjected to immunoprecipitation. J,K) Direct binding of rFGFR1 or rVEGFR3 to rMCB1 using a GST pull‐down assay.

The introduction of p53 did not influence MCB1 overexpressing‐reduced cell apoptosis upon targeted drugs (Figure S8A, Supporting Information), suggesting that MCB1 limits the response of HCC cells to targeted drugs through a p53‐independent mechanism. Intriguingly, the phosphorylation levels of STAT3, AKT, and ERK were significantly increased when MCB1 was knocked down and were significantly decreased upon MCB1 overexpression (**Figure** **5**A; Figure S8B, Supporting Information). Considering that the targets of sorafenib and lenvatinib are multiple receptor tyrosine kinases (RTKs), which are upstream regulators of STAT3, AKT and ERK, we speculated that MCB1 might influence the expression of RTKs. Human RTK phosphorylation antibody array revealed that MCB1 silencing resulted in an increase in 38 kinases and that MCB1 overexpression led to a decrease in 54 kinases (Table S7 and S8, Supporting Information). Among the overlapping kinases, fibroblast growth factor receptor 1 (FGFR1) and vascular endothelial growth factor receptor 3 (VEGFR3) and rearranged during transfection (RET) were sorafenib and lenvatinib targets (Figure 5B). Western blot analysis validated that the protein expression of FGFR1 and VEGFR3 was regulated by MCB1, while their mRNA levels were not influenced (Figure 5C; Figure S8C, Supporting Information). Upregulation of FGFR1 and VEGFR3 expression in the MCB1‐deficient hepatoma cells was further confirmed by immunofluorescence staining (Figure 5D,E). Consistently, ectopic MCB1 expression downregulated the protein levels of FGFR1 and VEGFR3 but not their mRNA levels (Figure S8D,E, Supporting Information).

Moreover, the proteasome inhibitor MG132 rather than the protein synthesis inhibitor CHX blocked the FGFR1 and VEGFR3 alteration by MCB1, suggesting that MCB1 promotes the proteasomal degradation of FGFR1 and VEGFR3 rather than synthesis (Figure 5F; Figure S8F, Supporting Information). Accordingly, depletion of MCB1 in hepatoma cells resulted in the accumulation of FGFR1 and VEGFR3, as well as ubiquitinated FGFR1 and ubiquitinated VEGFR3 (Figure 5G,H). The interactions between endogenous FGFR1 or VEGFR3 and MCB1 were confirmed by co‐IP experiments (Figure S8G, Supporting Information). To reveal the motif of MCB1 required for FGFR1 or VEGFR3 binding, we transfected various MCB1 truncations into HCCLM3 cells. Our data showed that FGFR1 and VEGFR3 were coimmunoprecipitated with the C‐terminus of MCB1 (Figure 5I). Importantly, purified GST‐MCB1 and recombinant FGFR1 or VEGFR3 were subjected to a GST bead pull‐down assay, and the results suggested that FGFR1 and VEGFR3 directly bind to MCB1 (Figure 5J,K).

### 2.8 FGFR1 and VEGFR3 Are Responsible for MCB1‐Mediated Targeted Drugs Resistance

**Figure 6:**
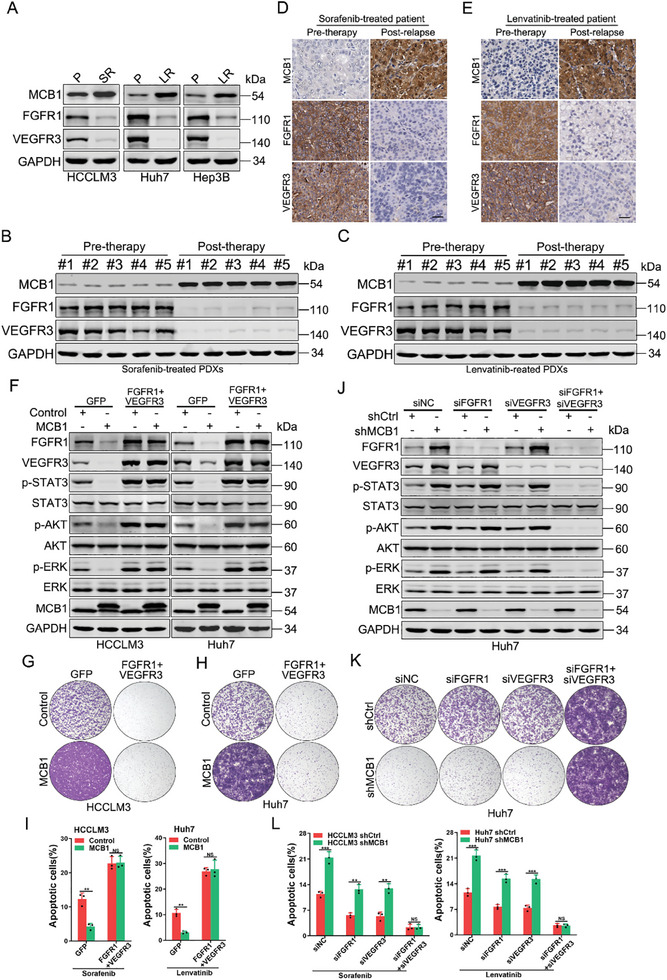
MCB1 regulates the targeted drug response by downregulating FGFR1 and VEGFR3 expression. A) Western blot analysis of indicated proteins in HCCLM3/Huh7/Hep3B and HCCLM3SR/Huh7LR/Hep3BLR cells. B) Western blot analysis of indicated proteins in sorafenib pre‐therapy and post‐therapy PDXs. C) Western blot analysis of indicated proteins in lenvatinib pre‐therapy and post‐therapy PDXs. D, E) Immunostaining of MCB1, FGFR1, and VEGFR3 in matched human HCC taken before sorafenib/lenvatinib therapy (Pre‐therapy) and after sorafenib/lenvatinib resistance (Post‐relapse) Scale bar, 25 µm. F) MCB1 overexpression cells and control hepatoma cells infected with lentivirus expressing FGFR1/VEGFR3 or control virus were subjected to western blot analysis. G) Colony formation assay of HCCLM3 MCB1 and control cells infected with lentivirus expressing FGFR1/VEGFR3 upon sorafenib treatment (1.25 µm) in a 12‐well dish for 10 days. H) Colony formation assay of Huh7 MCB1 and control cells infected with lentivirus expressing FGFR1/VEGFR3 upon lenvatinib treatment (0.625 µm) in a 12‐well dish for 10 days. I) MCB1 overexpression cells and control hepatoma cells infected with lentivirus expressing FGFR1/VEGFR3 were treated with targeted drugs for 48 h followed by cytometry analysis of apoptosis (*n* = 3). Data are presented as mean ± SD. J) MCB1 knockdown cells and control hepatoma cells were transfected with siFGFR1, siVEGFR3, or siNC for 48 h and then subjected to western blot analysis. K) Colony formation assay of Huh7 shMCB1 and control cells transfected with siFGFR1, siVEGFR3, or siNC with lenvatinib treatment (0.625 µm) in a 12‐well dish for 10 days. L) MCB1 knockdown cells and control hepatoma cells transfected with siFGFR1, siVEGFR3, or siNC were treated with targeted drugs for 48 h followed by cytometry analysis of apoptosis (*n* = 3). Data are presented as mean ± SD. Unless otherwise indicated, p‐values were determined by unpaired student's *t*‐test (two‐tail) and ^**^, ^***^, NS indicate *p*‐value < 0.01, < 0.001, not significant, respectively.

Notably, the expression of MCB1 was increased and FGFR1 or VEGFR3 was decreased in HCC cells resistant to targeted drugs (**Figure** **6**A). Consistently, low expression of FGFR1 or VEGFR3 was observed in the targeted drug‐resistant PDXs with high expression of MCB1 (Figure 6B,C; Figure S9A,B, Supporting Information) and in clinically relapsed tumors after targeted drugs therapy (Figure 6D,E). We then explored whether FGFR1 and VEGFR3 were functionally involved in MCB1‐mediated targeted drug resistance. Restoration of FGFR1 and VEGFR3 resensitized MCB1‐overexpressing cells to targeted drugs and recovered downstream signaling (Figure 6F–I; Figure S9C–E, Supporting Information). Conversely, concurrent knockdown of FGFR1 and VEGFR3 recapitulated the targeted drug‐resistant phenotype and abolished downstream signaling in MCB1 knockdown hepatoma cells, while knockdown of either of them showed a limited effect (Figure 6J–L; Figure S9F–J, Supporting Information). Taken together, the genetic data indicate that FGFR1 and VEGFR3 are responsible for MCB1‐mediated sorafenib and lenvatinib resistance.

### 2.9 Targeting MCB1 Restores Sorafenib and Lenvatinib Response in HCC

**Figure 7:**
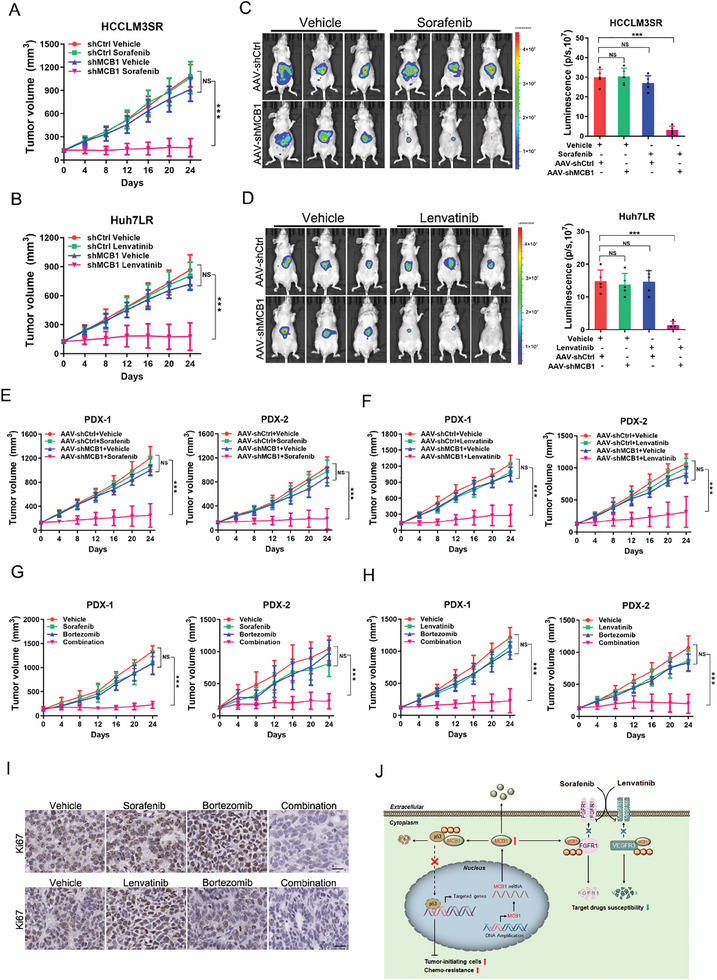
Targeting MCB1 restores the response of targeted drugs in HCC. A) Tumor growth curves of mice after the subcutaneous injection of HCCLM3SR shMCB1 and control cells and intraperitoneal administration of sorafenib, *n* = 5 for each group. Data are presented as mean ± SD. B) Tumor growth curves of mice after the subcutaneous injection of Huh7LR shMCB1 and control cells and intraperitoneal administration of lenvatinib, *n* = 5 for each group. Data are presented as mean ± SD. C) Nude mice were orthotopically (sub‐capsular space of the liver) xenografted with HCCLM3SR‐luc cells (1 × 10^6^ cells) and treated intravenously with AAV‐shCtrl or AAV‐shMCB1 and orally with vehicle or sorafenib daily (30 mg/kg) (*n* = 5 each group). Representative bioluminescent images of mice in indicated groups are shown. Data are presented as mean ± SD. D) Nude mice were orthotopically (sub‐capsular space of the liver) xenografted with Huh7LR‐luc cells (1 × 10^6^ cells) and treated intravenously with AAV‐shCtrl or AAV‐shMCB1 and orally with vehicle or lenvatinib daily (4 mg/kg) (*n* = 5 each group). Representative bioluminescent images of mice in indicated groups are shown. Data are presented as mean ± SD. E) PDXs derived from the primary HCCs (Patient #9‐10) treated intratumorally with AAV‐shCtrl or AAV‐shMCB1 and orally with vehicle or sorafenib daily (30 mg/kg) (*n* = 5 each group). The xenograft growth was monitored. Data are presented as mean ± SD. F) PDXs derived from the primary HCCs (Patient #9‐10) treated intratumorally with AAV‐shCtrl or AAV‐shMCB1 and orally with vehicle or lenvatinib daily (4 mg/kg) (*n* = 5 each group). The xenograft growth was monitored. Data are presented as mean ± SD. G) PDXs derived from the primary HCCs (Patient #9‐10) treated intraperitoneal injection with Bortezomib (2 mg/kg, once a day) and orally with vehicle or sorafenib daily (30 mg/kg) (*n* = 5 each group). The xenograft growth was monitored. Data are presented as mean ± SD. H) PDXs derived from the primary HCCs (Patient #9‐10) treated intraperitoneal injection with Bortezomib (2 mg/kg, once a day) and orally with vehicle or lenvatinib daily (4 mg/kg) (*n* = 5 each group). The xenograft growth was monitored. Data are presented as mean ± SD. I) PDXs derived from indicated patients treated with Bortezomib or targeted drugs were subjected to Ki67 staining. Representative views were shown. Scale bar, 25 µm. J) Schematic model of the mechanism underlying amplified MCB1‐drived HCC initiation and drug resistance. Data are presented as mean ± SD. Unless otherwise indicated, *p*‐values were determined by unpaired student's *t*‐test (two‐tail) and ^***^, NS indicate *p*‐value < 0.001, not significant, respectively.

Importantly, we found that MCB1 interference sensitized targeted drug‐resistant HCC cells to growth inhibition and apoptosis induced by targeted drugs (**Figure** **7**A,B; Figure S10A–C, Supporting Information). To assess the therapeutic significance of MCB1 in HCC with drug resistance in vivo, an orthotopic (sub‐capsular space of the liver) xenograft model established using HCCLM3SR‐luc and Huh7LR‐luc cells was administrated with an adeno‐associated virus (AAV) targeting MCB1. Our data showed that the sensitivity of HCCLM3SR‐luc xenografts to sorafenib treatment was significantly restored by therapeutic AAV‐shMCB1 (Figure 7C), and so did the Huh7R‐luc xenografts to lenvatinib treatment (Figure 7D). No significant body weight change was observed among different groups during treatment (Figure S10D, Supporting Information). MCB1 knockdown, which was mediated by AAV, and the upregulation of FGFR1 and VEGFR3 were confirmed by western blot (Figure S10E,F, Supporting Information). In addition, PDX models were established using sorafenib or lenvatinib‐resistant patient HCCs (patient #9‐10) to evaluate the therapeutic potential of MCB1 blockade. MCB1 knockdown by AAV resensitized the PDXs to sorafenib or lenvatinib treatment (Figure 7E,F), accompanied with increased expression of FGFR1 or VEGFR3 in the post‐treatment tumors (Figure S10G,H, Supporting Information). No significant weight change of mice was observed during treatment (Figure S10I,J, Supporting Information).

As MCB1 is a subunit of the 26S proteasome, which has UIM motifs that recognize polyubiquitin chains and mediate the degradation of ubiquitin conjugates, proteasome inhibitor bortezomib was investigated in vivo. A notable growth suppression was observed when targeted drug‐resistant PDXs were treated with bortezomib plus targeted drugs, whereas neither alone led to growth inhibition (Figure 7G–I). The weight of mice did not change notably during treatment (Figure S10K,L, Supporting Information), which suggested that MCB1 could serve as a potential therapeutic target to overcome targeted drug resistance in HCC. Taken together, our results demonstrate that oncofetal MCB1 drives liver cancer initiation and determines the cell response to chemotherapeutics through distinct mechanisms (Figure 7J).

## 3 Discussion

In the present study, we discovered that MCB1 could be a novel liver oncofetal protein, which was highly expressed in the fetal liver, downregulated in adult liver, and reactivated in human HCC. This distinct expression pattern has clinical importance in HCC diagnosis and treatment.^[^ ^18^, ^19^ ^]^ Moreover, the expression of MCB1 in HGDNs suggests its value in early HCC screening in the high‐risk population, which is worthy of future investigation. Inspired by the emergence of MCB1 in HGDNs, we speculate that MCB1 might have a role in HCC initiation. Using a classic chemical‐induced HCC initiating model, we found a key role for MCB1 in HCC initiation. Furthermore, we clarified that MCB1 is not only highly expressed in liver T‐ICs but also notably enhances T‐IC properties. Through RNA sequencing, we found that p53 signaling was involved in MCB1‐mediated HCC onset. Given that p53 was reported to be involved in the repression of T‐ICs,^[^ ^20^ ^]^ the regulatory signaling network of p53 remains incomplete to date. Using multiple approaches, we confirmed that UIMs of MCB1 directly bound to the central DNA‐binding domain of p53 and thus modulated p53 degradation. Importantly, our data revealed that MCB1‐mediated p53 protein degradation led to hepatic T‐IC generation and liver cancer development. Our findings not only provide a novel mechanism of p53 inactivation in HCC cells but also demonstrate the crucial role of MCB1 in HCC development, which suggests that targeting MCB1 could be a novel therapeutic strategy for HCC patients.

TACE is the main treatment for patients with advanced HCC, but only a small proportion of those who receive TACE exhibited significant survival benefits. CDDP, doxorubicin, and 5‐FU are the most commonly used chemotherapeutic drugs in TACE treatment for HCC.^[^ ^21^ ^]^ Herein, we demonstrated that the MCB1‐overexpressing HCC cells were resistant to CDDP, and MCB1/p53 axis determines the CDDP response. The HCC patient cohort, PDO, and PDX models were then used, and the results suggested that the patients with low MCB1 levels had superior survival upon TACE treatment. Therefore, distinguishing patients sensitive to TACE therapy by evaluating MCB1 expression levels may be advisable and worthy of further clinical validation.

Sorafenib and lenvatinib are small‐molecule inhibitors of multiple receptor tyrosine kinases and are first‐line therapeutic drugs for advanced HCC patients.^[^ ^5^, ^22^ ^]^ Nevertheless, only a small proportion of HCC patients exhibit satisfactory therapeutic effects. Thus, identifying biomarkers that can be used to predict the response of HCC patients to targeted drugs is urgently needed. The previous in vitro biochemical kinase assay demonstrated that VEGFRs, PDGFR, FGFRs, c‐MET, RET, Flt3, c‐KIT, and Raf kinase could be targeted by sorafenib.^[^ ^23^ ^]^ Nevertheless, the expression of RAF was found to be lower in HCC than in paracarcinoma tissue.^[^ ^24^ ^]^ VEGFR and FGFR were reported to be associated with the sorafenib response in HCC.^[^ ^25^, ^26^ ^]^ In the current study, we demonstrated that MCB1 bound directly to FGFR1 or VEGFR3 and promoted the degradation of ubiquitinated FGFR1 or VEGFR3. More importantly, our data showed that the interference of MCB1 increased FGFR1 and VEGFR3 activation and sensitized hepatoma cells to targeted drugs, suggesting that the interaction between MCB1 and FGFR1 or VEGFR3 determines the HCC cell response to sorafenib and lenvatinib. Studies using patient cohorts, PDOs, and PDXs further suggested that MCB1 could be a novel biomarker for personalized therapy with targeted drugs.

Since sorafenib could inhibit post‐TACE hypoxia‐induced angiogenesis in HCCs, TACE plus sorafenib should be a synergistic combination. However, the results of clinical trials assessing the combination of TACE and sorafenib in HCC patients were inconsistent, with 3 negative trials^[^ ^27^, ^28^, ^29^ ^]^ and only 1 positive trial.^[^ ^30^ ^]^ Considering that MCB1 silencing sensitized HCC cells to either TACE or targeted drugs, we next explored whether MCB1 was associated with the therapeutic effect of TACE plus sorafenib. Survival analysis of patients who received both postsurgical TACE and sorafenib showed that low MCB1 expression in HCC tissues was correlated with longer OS, suggesting that MCB1 could be used as a potential marker for the selection of patients for TACE plus sorafenib, which has clinical importance in the current practice of HCC treatment.

Due to the diverse etiologies and complex pathogenesis, HCC is a highly heterogeneous malignant tumor, a large proportion of which possesses targeted drug resistance.^[^ ^31^, ^32^, ^33^ ^]^ Elucidating the molecular mechanism of targeted drug resistance and developing novel therapeutic strategies was expected to improve the clinical benefits of targeted drugs for patients.^[^ ^34^, ^35^, ^36^ ^]^ Our results demonstrate that MCB1 suppression by AAV‐shMCB1 resensitized the HCC cells with resistance to targeted drugs in both orthotopic xenografts and PDX models, suggesting that targeting MCB1 could be a promising strategy for targeted drugs‐resistant HCC. The ubiquitin‐proteasome pathway is involved in cell dedifferentiation, stress response, and cell cycle control.^[^ ^37^ ^]^ Targeting the proteasome machinery with small molecules has been successfully translated into clinical cancer therapies.^[^ ^38^ ^]^ In the current study, we also provide evidence for using a proteasome inhibitor bortezomib to overcome MCB1‐mediated resistance and restore the targeted drug response. These data reinforce the concept that intervention of specific resistance mechanisms could overcome targeted drug resistance in clinic.

In conclusion, our findings demonstrated that oncofetal MCB1 might not only act as a driver for HCC initiation and chemoresistance but also serve as a serum biomarker for individualized HCC therapy, which is worthy of further validation by clinical trials.

## 4 Experimental Section

### Patients and Analysis

High‐grade dysplastic nodules (HGDNs) were confirmed by postoperative pathological examination. The HCC tumor and adjacent nontumor tissues (precancerous normal liver tissue) from cohort 1 (n = 160) were obtained from patients undergoing surgical resections at Eastern Hepatobiliary Surgery Hospital (EHBH) from 2006 to 2010. Detailed clinicopathological features of the patients in cohort 1 are described in Table S2, supporting information. Cohort 1 is followed for at least 60 months. Overall survival (OS) was defined as the interval between the dates of surgery and death. Patient disease‐free survival (DFS) was defined as the time interval between the dates of surgery and recurrence; if recurrence was not confirmed, patients were censored on the date of death or the last follow‐up.

### TACE Cohort

To analyze the factors associated with TACE benefits in patients after hepatectomy, a total of 173 patients with radical hepatectomy from EHBH were enrolled. Of them, 88 patients received TACE therapy after curative resection (TACE group, cohort 2). The other patients without TACE therapy were included in the control group. The detailed clinicopathological features are described in Table S3 (Supporting Information). The regimen for preventive adjuvant TACE consisted of 0.75 g 5‐fluorouracil, 60 mg CDDP, and an emulsion mixed with 16 mg mitomycin C and 5 mL of lipiodol.

### Multicenter Sorafenib Cohort

To analyze the factors associated with sorafenib benefit in patients with relapsed HCC, a total of 332 patients with HCC recurrence after surgery from three hospitals [Eastern Hepatobiliary Surgery Hospital (EHBH) in Shanghai; Sun Yat‐sen University (SYSU) Cancer Center in Guangzhou; and The Third Affiliated Hospital of SYSU in Guangzhou] were enrolled. Of them, 159 patients received sorafenib therapy after recurrence (sorafenib group, cohort 3). The other patients without sorafenib therapy were included in the control group. Their detailed clinicopathologic features are described in Table S4 (Supporting Information). Sorafenib was given to patients at 400 mg twice a day. Treatment interruptions and up to two dose reductions (200 mg twice a day and 200 mg per day.) were permitted for drug‐related adverse effects.

### Lenvatinib Cohort

To analyze the factors associated with lenvatinib benefit in patients with relapsed HCC, a total of 68 patients with HCC recurrence after surgery from EHBH were enrolled. Of them, 31 patients received lenvatinib therapy after recurrence (lenvatinib group, cohort 4). The other patients without lenvatinib therapy were included in the control group. The detailed clinicopathological features are described in Table S5 (Supporting Information). Lenvatinib was given at 12 mg per day (body weight > 60 kg) or 8 mg per day (body weight < 60 kg).

### TACE and Sorafenib Cohort

To analyze the factors associated with TACE and sorafenib benefits in patients after hepatectomy, a total of 169 patients with radical hepatectomy from EHBH were enrolled. Of them, 83 patients received TACE and sorafenib therapy after curative resection (TACE and sorafenib group, cohort 5). The other patients who did not receive TACE and sorafenib therapy were classified into the control group. The detailed clinicopathological characteristics are shown in Table S6 (Supporting Information).

In all of the above cohorts, informed consent was obtained before recruitment, and the study protocols were approved by the Ethics Committee of the EHBH, Sun Yat‐sen University (SYSU) Cancer Center, or No. 3 Affiliated Hospital of SYSU. Immunohistochemical assessments of the above cohorts were based on sections from primary tumors.

### Cell Lines and Lentivirus

Human normal hepatocyte THLE‐3 was purchased from ATCC and cultured in BEGM from Lonza. The normal liver cell lines HL7702 were obtained from the Cell Bank of the Type Culture Collection of the Chinese Academy of Sciences (Shanghai Institute of Cell Biology of the Chinese Academy of Sciences). For adhesion culture, cells were seeded in normal plates or dishes, and cultured in Dulbecco's modified Eagle medium (DMEM, Invitrogen, USA) with 10% fetal bovine serum (FBS, GE Healthcare Life Sciences, USA) and 1% penicillin/streptomycin (HyClone, USA) at 37 °C incubator with 5% CO_2_. An MCB1 knockdown lentiviral vector (designated as shMCB1) was generated using the BLOCK‐iT Lentiviral miR RNAi Expression System (Invitrogen) according to the manufacturer's instructions. Lentiviral interference vectors were constructed by LR recombined reaction between pENTR/U6 entry plasmid and pLenti6/BLOCKiT lentiviral expression plasmid. Lentiviral miR empty vector was used as a control (designated as shCtrl). An MCB1‐overexpressing lentiviral vector was constructed by inserting the human MCB1 gene into the pLenti6/V5‐DEST vector (Invitrogen) (designated as MCB1). A vector expressing green fluorescent protein (GFP) was used as a negative control (designated as Control). Genomic plasmids were cotransfected with the lentiviral packaging plasmids into HEK‐293 cells to pack the lentiviral particles, and the particles were further amplified and purified. shMCB1 and MCB1 stable transfectants and scramble control transfectants were established using the corresponding packaged lentivirus.

In order to establish sorafenib‐resistant or lenvatinib‐resistant cell lines, HCCLM3 was incubated with sorafenib (Selleck, S7397) and Huh7 or Hep3B cells were incubated with lenvatinib (Selleck, S1164) at a concentration slightly lower than their IC_50_, with the drug concentration being elevated by 0.2 µM per week for 6 to 7 months. Sorafenib‐resistant HCCLM3 cells were established and named HCCLM3SR, and Huh7 and Hep3B cells with lenvatinib resistance were obtained and named Huh7LR and Hep3BLR. These sorafenib‐resistant or lenvatinib‐resistant cell lines were cultured in the presence of sorafenib or lenvatinib after establishment to avoid the loss of resistance.

### Data Analysis

Statistical analysis was performed using SPSS V.18.0. The data are expressed as the mean ± SD. Two‐tailed Student's *t*‐test or the Mann‐Whitney U test was used to compare two continuous variables, and the chi‐square test was used to compare qualitative variables. The DFS and OS of distinct subgroups were compared by the Kaplan‐Meier method and log‐rank analysis. Pearson's correlation analysis was performed to determine the correlation between two variables. For IHC staining density analysis, the median score was set as the cutoff value to divide the cohort into high and low subgroups. Each dataset was analyzed separately. A *p*‐value <0.05 was considered statistically significant.

## Conflict of Interest

The authors declare no conflict of interest.

## Author Contributions

D.‐M.X., J.‐Y.L., and Y.‐C.W. contributed equally to this work. D.‐M.X., J.‐Y.L., and Y.‐C.W. conducted all experiments and analyzed the data. W.D. and L.X. provided clinical samples. W.D. provided pathology evaluation and D.‐M.X. analyzed clinical data. D.‐T.H., T.‐L. Z., C.Z., W.‐Q.J., X.‐J.L., Y.‐H.S. J.Z., H.L., H.‐Y.L., and W.S. provided support with experimental techniques. D.‐M.X. wrote the manuscript and J.D. contributed to the revision. J.D. conceived the project.

## Supporting information

Supporting Information

## Data Availability

Sequencing data have been deposited at NCBI BioProject under accession number PRJNA826069. All other data supporting the findings of this study are available from the corresponding author on reasonable request.
