# Supplementary material for: Oncofetal MCB1 Is a Functional Biomarker for HCC Personalized Therapy
Source: Adv Sci (Weinh). 2024 Oct 14;11(45):2401228. doi: 10.1002/advs.202401228 (PMC11615823; doi:10.1002/advs.202401228)
Supplement: Supplementary file 1 — Supporting Information [file ADVS-11-2401228-s001.docx]

Oncofetal MCB1 is a Functional Biomarker for HCC Personalized Therapy

Daimin Xiang^1,2,3,#^, Junyu Liu^1,#^, Yichuang Wang^1,#^, Dingtao Hu^1^, Cheng Zhang^4^, Tanlun Zeng^1^, Weiqi Jiang^4^, Xijun Liang^1^, Wei Dong^5^, Wen Sun^4^, Li Xu^6^, Hengyu Li^7^, Yihai Shi^8^, Jian Zhang^9^, Hui Liu^10^, Jin Ding^1,4,*^

^1^Clinical Cancer Institute, Center for Translational Medicine, Naval Military Medical University, Shanghai, 200433, China;

^2^Medical Innovation Center, Shanghai East Hospital, School of Medicine, Tongji University, Shanghai, 200120, China;

^3^Institute of Hepatobiliary and Pancreatic Surgery, Department of Hepatobiliary and Pancreatic Surgery, Shanghai East Hospital, School of Medicine, Tongji University, Shanghai, 200120, China;

^4^National Center for Liver Cancer, Naval Military Medical University, Shanghai, China;

^5^Department of Pathology, Third Affiliated Hospital of Naval Military Medical University, Shanghai, 200438, China;

^6^Department of Liver Surgery, Sun Yat-sen University Cancer Center, Collaborative Innovation Center for Cancer Medicine, Guangzhou, 510060, China;

^7^Department of Breast and Thyroid Surgery, Changhai Hospital, Naval Military Medical University, Shanghai 200433, China;

^8^Department of Gastroenterology, Shanghai Pudong New Area Gongli Hospital, Shanghai 200135, China;

^9^The State Key Laboratory of Cancer Biology, Department of Biochemistry and Molecular Biology, The Fourth Military Medical University, Xi’an 710032, China;

^10^Department of Hepatic Surgery, Third Affiliated Hospital of Naval Military Medical University, Shanghai, 200438, China.

^#^The authors have contributed equally to this work.

***Correspondence author address:**

Dr. Jin Ding (dingjin1103@163.com). Clinical Cancer Institute, Center for Translational Medicine, Naval Military Medical University, Shanghai, 200433, China Tel: 86-21-81870801.

**Experimental Section**

*Plasmids and recombinant protein expression:*Flag-MCB1, Flag-MCB1 N-terminal mutant and Flag-MCB1 C-terminal mutant (MCB1 Flag, MCB1 MUN and MCB1 MUC, respectively), His-p53 and plasmids expressing His-p53 mutants (p53 1-365AA, p53 1-325AA, p53 1-292AA, p53 Δ95-292AA, p53 62-393AA and p53 95-393AA), and plasmids expressing exogenous HA-tagged ubiquitin (HA-Ub) were obtained from OBiO Technology (Shanghai) Corp., Ltd. JetPEI DNA transfection reagent (Polyplus) was used for transient transfection of plasmids into cell lines according to the manufacturer’s instructions. For recombinant human MCB1 fused with GST (GST-MCB1), the MCB1 gene was cloned into the pMKH vector for GST-fusion proteins. The p53 gene and FGFR1 gene were cloned into the pIRES2-eGFP vector and fused with His tags for mammalian expression. All constructs were verified by DNA sequencing analysis. The rp53 and rFGFR1 were expressed by *Escherichia coli* BL21 and purified using glutathione Sepharose beads (Sigma). GST-MCB1 was expressed by *E. coli* BL21 cells and purified using MBP beads (Sigma).

*GST pull-down assay:* For the GST pull-down assay, 3 μg of purified GST-MCB1 and 3 μg of rp53, rFGFR1 or rVEGFR3 were incubated with 20 μL of GST beads (Sigma-Aldrich, A0512) for 2 h in PBS at 4 ℃. After extensive washing, the samples were suspended in reducing SDS loading buffer, boiled for 5 min, and subjected to SDS-PAGE followed by immunoblotting. The primary antibodies used included anti-GST (1:1000, Sigma-Aldrich, M6295) and His tag (1:500, Proteintech, 66005-1-1g).

*Mice and HCC induction:* C57BL/6-Gt (ROSA)26Sor^tm1(MCB1)Smoc^ mice (MCB1 knock-in mice), C57BL/6-MCB1^flox/flox^ mice and Alb-Cre transgenic mice were obtained from the Nanjing Model Organisms Center, Inc. Mice were maintained under a 12 h light/12 h dark cycle at 25 °C and given food and water ad libitum. Briefly, MCB1 knock-in (KI) mice were constructed by inserting both an upstream ‘stop signal’ containing lox-stop-lox sequences and a downstream MCB1 gene at the Rosa26 locus (Rosa26-LSL-MCB1). The MCB1 KI mice and MCB1^flox/flox^ mice were backcrossed to a C57BL/6 background at least 10 times. Through mating with Alb-Cre+ mice, liver-specific MCB1 transgenic mice (MCB1-TG mice, ROSA26^MCB1^; Alb-Cre+/-) and liver-specific knockout mice (MCB1^hep-/-^mice, MCB1^flox/flox^; Alb-Cre+/-) were generated. ROSA26^MCB1^; Alb-Cre-/- mice and MCB1^flox/flox^, Alb-Cre-/- mice were used as controls. For HCC induction, male mice were intraperitoneally injected with a single dose of diethylnitrosamine (DEN) (25 mg/kg body weight) at 14 days of age. At four weeks of age, mice were then intraperitoneally injected with carbon tetrachloride (CCl_4_) (0.5 ml/kg in olive oil, Shanghai Macklin Biochemical Co., Ltd.) twice a week for up to 8 weeks and were sacrificed at the age of ~20 weeks. Adeno-associated virus overexpressing p53 (AAV-p53) or control was injected. Mouse livers and serum were collected for subsequent experiments.

*Xenografted tumor formation and PDX model:* For the *in vivo* transformation assay, MCB1 or control stably transfected HL7702 cells were digested into single cells and mixed with Matrigel (1:1). The mixture was injected subcutaneously into eight NOD-SCID mice at 1×10^3^ cells per mouse. Xenografted tumor formation was monitored, and the mice were euthanized 10 weeks post-inoculation.

For the patient-derived xenograft (PDX) model, primary tumor samples were obtained for xenograft establishment as described previously ^[1]^. When the PDX volume reached approximately 100 mm^3^, the mice were randomly assigned into different groups as indicated. Mice in the CDDP group were intratumorally injected with CDDP (2 g/kg) or vehicle every two days for up to 16 days (n=5 for each group). Mice in the sorafenib group were intraperitoneally injected with sorafenib (30 mg/kg) or vehicle daily for 24 days (n=5 for each group). Mice in the lenvatinib group were intraperitoneally injected with lenvatinib (4 mg/kg) or vehicle daily for 24 days (n=5 for each group). Mice in the bortezomib group were intraperitoneally injected with bortezomib (0.5 mg/kg) or vehicle three times a week for 24 days (n=5 for each group). Tumor volumes were measured by caliper twice a week using the formula Volume=π/6*L*W^2^, where L is the longest tumor axis and W is the shortest tumor axis. When the PDX volume reached approximately 1500 mm^3^, the mice were euthanized by CO_2_, and the tumors were sectioned or frozen for subsequent analysis.

For orthotopic (sub-capsular space of the liver) xenograft model, a skin incision of ~2.0 cm was made abdomen in the region of the liver of anesthetized mice. 1×10^6^ luciferase-labeled HCCLM3SR or Huh7LR cells mixed with Matrigel (BD Biosciences, USA) were injected into the sub-capsular space of the liver. The xenograft growth was measured weekly by bioluminescent imaging technology (Xenogen IVIS 100 Imaging System). When the sizes of xenografts were matched, the mice were randomized for intravenous injection of AAV-shMCB1 (50 nmol, once), or administration of sorafenib (30 mg/kg) or lenvatinib (4 mg/kg, oral average), or combination treatment for 24 days (n=5 each group, randomized allocated). All animal procedures were consistent with animal welfare recommendations, and protocols were approved by the Ethics Committee of EHBH.

*Tissue dissociation and organoid culture:* Organoid culture was performed as previously described.^[2]^ Fresh liver cancer tissue was obtained, the blood, fat, necrotic and connective tissue on the tissue were cut off, and the area with abundant tumor cells was preserved and cut into pieces. The tissues were placed in 5 mL of 5 mM PBS/EDTA liquid for 15 min at room temperature. Then, the tissues were placed in 5 mL of 1 mM PBS/EDTA containing 2 × TrypLe and digested at 37 ℃ for 1 hour. The cells are suspended in liquid by mechanical force blowing away the tissue mass. Isolated cells were collected in DMEM/F12 medium at 4 °C, centrifuged at 300 g for 5 min and regrouped into granular cells. The granular cells suspended in 120 μL of GFR Matrigel were seeded in 24 or 48 well cell culture plates. The drop was solidified by a 30-minute incubation at 37 °C and 5% CO2. After solid drops formed, 1.5 mL of the organoid culture media was added to the well, and the medium was changed every 3-4 days. CDDP, sorafenib or bortezomib at different concentrations was added to the organs when they grew to a certain amount and size.

*Immunohistochemistry and immunofluorescence staining:* The tissue samples were fixed with 10% neutral formaldehyde, embedded in paraffin, and sectioned for hematoxylin-eosin (H&E) staining or immunohistochemical (IHC) staining as described previously.^[3]^ In brief, after antigen retrieval, sections or tissue microarrays (TMAs) were blocked with bovine serum antigen albumin (BSA) and incubated with the indicated primary antibody and then secondary antibody. A diaminobenzidine (DAB) colorimetric reagent solution was used, followed by hematoxylin counterstaining. The slides were scanned, and representative images were captured. IHC scoring was based on the percentage of positively stained cells, and staining intensity was assessed by Image Scope software (Aperio Technologies, Inc.). Briefly, the program automatically counted pixels and measured the intensity of positive (brown staining) pixels. The staining score for each sample was defined as average positivity (sum intensity of positive pixels per total positive pixels) times positivity rate (number of positive pixels per total pixels). The median score of MCB1 and p53 was set as cut-off value to divide the cohort into high and low subgroups.

Immunofluorescence staining was performed using a tyramide signal amplification (TSA) fluorescence kit (TSA Plus Fluorescein, NEL741001KT; PerkinElmer, Waltham, Massachusetts, USA) according to the manufacturer’s instructions. The antibodies used for IHC or immunofluorescence staining are listed in Supplementary table 9.

*Flow cytometric analysis:* The apoptosis of hepatoma cells was measured by flow cytometry using an APC Annexin V Apoptosis Detection Kit (BioLegend B287709). Briefly, 1×10^6^ cells were seeded in six-well plates and then treated with bortezomib, CDDP, sorafenib and lenvatinib for 48 hours. The cells were harvested and washed twice with cold cell staining buffer, resuspended in 100 μL of Annexin V binding buffer, and then incubated with 5 μL of APC Annexin V and 5 μL of 7-AAD viability staining solution for 15 minutes at room temperature in the dark. The cell suspension was then incubated with 400 μL of Annexin V binding buffer followed by flow cytometry analysis.

*RNA interference:* Small interfering RNAs (siRNAs) were synthetized by GenePharma (Shanghai, China). The siRNA target sequences are listed in Supplementary table10. The siRNAs were transfected into hepatoma cells at a final concentration of 200 nM using Lipofectamine 2000 Transfection Reagent according to the manufacturer’s instructions (Thermo Fisher Scientific). The cells were harvested or subjected to further downstream experiments 24-72 hours after transfection.

*RNA sequencing:* Total RNA (MCB1-TG, MCB1^hep-/-^ and their WT control liver cancer tissues) was extracted using the mirVana miRNA Isolation Kit (Ambion) following the manufacturer’s protocol. RNA integrity was evaluated using the Agilent 2100 Bioanalyzer (Agilent Technologies, Santa Clara, CA, USA). The samples with an RNA integrity number (RIN) ≥ 7 were subjected to subsequent analysis. The libraries were constructed using the TruSeq Stranded mRNA LT Sample Prep Kit (Illumina, San Diego, CA, USA) according to the manufacturer’s instructions. Then, these libraries were sequenced on the Illumina sequencing platform (HiSeqTM 2500 or Illumina HiSeq X Ten), and 125 bp/150 bp paired-end reads were generated.

Transcriptome sequencing and analysis were conducted by OE Biotech Co., Ltd. (Shanghai China). Raw data (ram reads) were processed using Trimmomatic. The reads containing poly-N and the low-quality reads were removed to obtain clean reads. Then, the clean reads were mapped to the reference genome using HISAT2. The FPKM value of each gene was calculated using cufflinks, and the read counts of each gene were obtained by htseq-count. DEGs were identified using the DESeq (2012) R package functions estimate Size Factors and nbinom Test. A P value < 0.05 and fold change > 2 or fold change < 0.5 were set as the thresholds for significant differential expression. Hierarchical cluster analysis of DEGs was performed to explore gene expression patterns. KEGG pathway enrichment analysis of DEGs was performed using R based on the hypergeometric distribution. Sequencing data have been deposited at NCBI BioProject under accession number PRJNA826069.

*Protein kinase chip array:* The Human RTK Phosphorylation Antibody Array was obtained from RayBiotech (Guangzhou) to simultaneously detect the relative levels of 71 phosphorylated human RTKs according to the manufacturer’s instructions. In brief, cell lysates of shMCB1 or MCB1 and control hepatoma cells were incubated with the antibody array membrane, and biotinylated anti-phosphotyrosine antibody was used to detect phosphorylated tyrosines on activated receptors. After HRP-streptavidin incubation, chemiluminescence signals were measured using an ImageQuant LAS 4000 Scanner (GE Healthcare Corporate). Data analysis was performed by RayBiotech. Raw data underwent background subtraction and positive control subtraction, and differentially expressed proteins were selected with fold change <0.8 or fold change >1.2.

*Enzyme-linked immunosorbent assay (ELISA):* Human blood plasma samples were collected from early HCC patients in EHBH and healthy controls in the physical examination center of EHBH. The protein level of MCB1 was determined by our own Human MCB1 ELISA Kit. In brief, MCB1 capture antibody was coated onto the plates at 4 °C overnight. The cells were washed with 1× washing buffer 3 times and then blocked with 1× assay buffer for 2 hours. The gradient concentrations of standard MCB1 protein (10, 5, 2.5, 1.25, 0.625, 0.3125, 0.1562, 0.0781 ng/mL) or patient plasma samples were added to each well and incubated with MCB1 detection antibody. Horseradish peroxidase (HRP) working solution and 3,3’,5,5’-tetramethylbenzidine (TMB) were added in sequence for coloring and ended in 1× stop solution. The OD value at 450 nm was measured under a microplate reader.

*Analysis of copy number by quantitative PCR:* The copy number changes in MCB1 were evaluated by qPCR using the qPCRTM MasterMix for SYBR Green, as previously reported ^[4]^. The qPCR reactions for each sample and each gene were performed in triplicate. Each copy number calculation was performed using the comparative Ct method ^[5]^. DNA from the normal tissue from each sample was used as the control ^[6]^; MCB1 gene copy number in the normal tissue was set as 2, and a copy number more than 4 was considered to be a gain, as in a previous study ^[7]^. The sequences of the primers used are listed in Supplementary table S10.

*Real-time PCR analysis:* Total RNA was extracted from tissues or cells using TRIzol (Invitrogen) and was reverse transcribed using a Reverse Transcription System (Promega) to synthesize cDNA. cDNA was mixed with a SYBR Green PCR Kit (Roche) and specific primers and underwent real-time PCR on a Roche Light Cycler 96 System (Roche, USA). PCR conditions included 1 cycle at 95 °C for 5 minutes, followed by up to 40 cycles of 95 °C for 15 seconds (denaturation), 60 °C for 30 seconds (annealing) and 72 °C for 30 seconds (extension). The sequences of the primers used are listed in Supplementary table S10. The specificity of the primers was confirmed by melting curves following the reaction. Each sample was measured in triplicate biological replicates. Each experiment was repeated at least three times.

*Western blot analysis:* Protein extracts of HCC cells or HCC tissues were quantified by BCA protein assay. Thirty micrograms of proteins were subjected to SDS-PAGE and then transferred to nitrocellulose membrane. The membrane was blocked with 5% non-fat milk and incubated with the primary antibody overnight. The protein band, specifically bound to the primary antibody, was detected using an IRDye 800CW-conjugated secondary antibody and LI-COR imaging system (LI-COR Biosciences). The antibodies used for Western blotting are listed in online Supplementary table S9.

*Immunoprecipitation (IP) analysis:* Hepatoma cells were harvested by IP lysis buffer with a protease inhibitor cocktail (Sigma-Aldrich, St. Louis, MO). The cell lysates were centrifuged at 4 °C at 12000 × g for 15 min, and the supernatant was quantified. The supernatant was incubated with the appropriate primary antibodies overnight, followed by incubation with protein A/G PLUS-Agarose beads (Santa Cruz Biotech., sc-2003) for 4 h. After three washes using ice-cold lysis buffer, the beads were boiled with 5x loading buffer for 5 min, followed by SDS-PAGE and western blotting using the appropriate primary and secondary antibodies.

*Proteasome activity assay:* The Proteasome-Glo Cell-Based Assay Kit (Promega) was used to assess proteasome activity as described previously^[1]^. Briefly, cells were seeded at 5×10^3^ cells per well into 96-well plates. Proteasome-Glo Cell-Based Reagents were added to the plate and incubated for 15 minutes. Luminescence was detected using the Synergy 2 Multi-Mode Reader (BioTek). The data were normalized to the cell number using the CellTiter-Glo Luminescent Cell Viability Assay (Promega).

*Colony formation assays:* A colony formation assay was used to assess the transformation activity of cells. Briefly, 1 mL of 0.66% soft agar solution was poured into a 12-well plate and solidified at room temperature. For the top layer, 1 mL of 0.33% agar solution containing 3×10^3^ cells with 10% FBS was added. An extra 1 mL of cell culture medium was added. The cells were cultured for three weeks, and the colonies formed were counted under a microscope.

*Spheroid formation assay:* Five hundred single HCC cells were counted and seeded in 96-well Ultra-Low Attachment Microplates (Corning, USA) and cultured in serum-free DMEM/F12 (Gibco) with 20 mg/mL bFGF (Invitrogen), 20 ng/ml EGF (Peprotech), 4 mg/mL insulin (Invitrogen) and B27 (1:50, Invitrogen) in a 37 °C incubator with 5% CO2. After 7 days of culture, the cells were photographed under a microscope.

*In vitro limiting dilution assay:* Gradient numbers of HCC cells (64, 32, 26, 8, 4, 2) with eight replicates were seeded in 96-well ultralow attachment plates and cultured for 7 days. Based on the frequency of wells with spheres forming, the proportion of tumor-initiating cells was determined using Poisson distribution statistics and the LCalc Version 1.1 software program (Stem Cell Technologies, Inc. Vancouver, Canada) ^[8]^.

*In vivo limiting dilution assay:* Cells in spheroid formation culture were digested into single cells, and the indicated numbers of cells (1×10^3^, 5×10^3^, 1×10^4^, 5×10^4^) were suspended in medium and mixed with Matrigel at a 1:1 ratio. Then, the cells were injected subcutaneously into NOD-SCID mice (n=4). Kinetic of tumor formation was evaluated per week for 8 weeks. Frequency of T-ICs was determined using ELDA software (http://bioinf.wehi.edu.au/software/elda/index.html) provided by the Walter and Eliza Hall Institute.

Reference:

[1] T.Y. Jiang, Y.F. Pan, Z.H. Wan, Y.K. Lin, B. Zhu, Z.G. Yuan, Y.H. Ma, Y.Y. Shi, T.M. Zeng, L.W. Dong, Y.X. Tan, H.Y. Wang, *Sci Transl Med* **2020**, 12,

[2] S.H. Lee, W. Hu, J.T. Matulay, M.V. Silva, T.B. Owczarek, K. Kim, C.W. Chua, L.J. Barlow, C. Kandoth, A.B. Williams, S.K. Bergren, E.J. Pietzak, C.B. Anderson, M.C. Benson, J.A. Coleman, B.S. Taylor, C. Abate-Shen, J.M. McKiernan, H. Al-Ahmadie, D.B. Solit, M.M. Shen, *Cell* **2018**, 173, 515.

[3] T. Han, D.M. Xiang, W. Sun, N. Liu, H.L. Sun, W. Wen, W.F. Shen, R.Y. Wang, C. Chen, X. Wang, Z. Cheng, H.Y. Li, M.C. Wu, W.M. Cong, G.S. Feng, J. Ding, H.Y. Wang, *J Hepatol* **2015**, 63, 651.

[4] J. Soh, N. Okumura, W.W. Lockwood, H. Yamamoto, H. Shigematsu, W. Zhang, R. Chari, D.S. Shames, X. Tang, C. MacAulay, M. Varella-Garcia, T. Vooder, Wistuba, II, S. Lam, R. Brekken, S. Toyooka, J.D. Minna, W.L. Lam, A.F. Gazdar, *PLoS One* **2009**, 4, e7464.

[5] K.J. Livak, T.D. Schmittgen, *Methods* **2001**, 25, 402.

[6] I.K. Kolasa, A. Rembiszewska, A. Felisiak, I. Ziolkowska-Seta, M. Murawska, J. Moes, A. Timorek, A. Dansonka-Mieszkowska, J. Kupryjanczyk, *Cancer Biol Ther* **2009**, 8, 21.

[7] W. Sun, S.C. Li, L. Xu, W. Zhong, Z.G. Wang, C.Z. Pan, J. Li, G.Z. Jin, N. Ta, W. Dong, D. Liu, H. Liu, H.Y. Wang, J. Ding, *Clin Cancer Res* **2020**, 26, 4302.

[8] D. Xiang, Z. Cheng, H. Liu, X. Wang, T. Han, W. Sun, X. Li, W. Yang, C. Chen, M. Xia, N. Liu, S. Yin, G. Jin, T. Lee, L. Dong, H. Hu, H. Wang, J. Ding, *Hepatology* **2017**, 65, 1566.


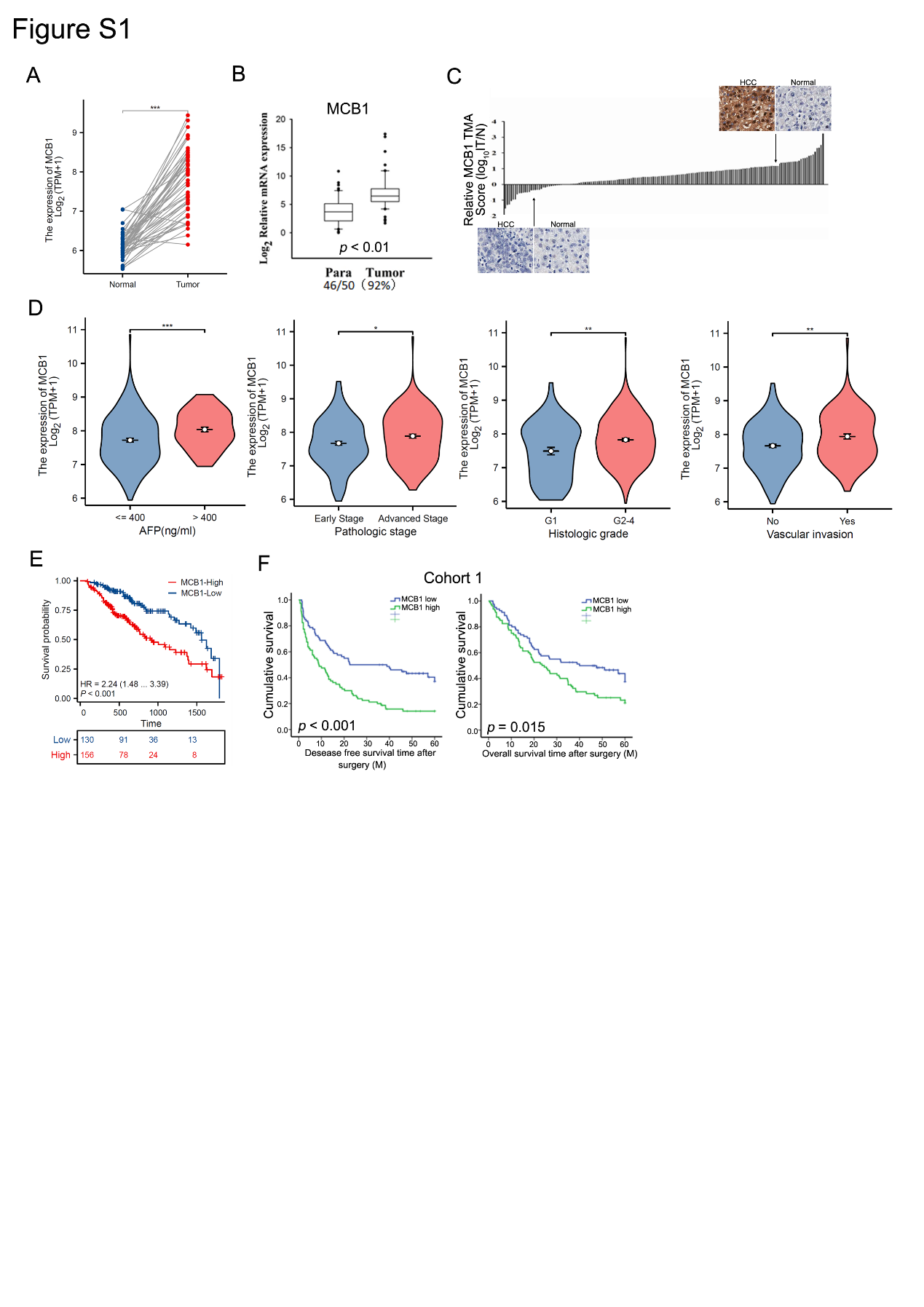


**Figure S1.** MCB1 predicts poor prognosis of HCC patients. A) MCB1 mRNA levels were higher in HCC tissues than in normal tissues in TCGA-LIHC dataset. P < 0.001 by two tailed t test. B) mRNA levels of MCB1 transcripts in 50 pairs of HCC samples were determined by real-time PCR. β-actin was used as a loading control. C) Representative views of IHC staining of MCB1 in human HCC and peritumoral normal tissues from cohort 1. The vast majority of patients (83.7%) exhibited increased MCB1 expression in their HCCs. D) MCB1 mRNA expression level was significantly elevated in patients with higher serum AFP level, advanced pathological stage, higher histological grade, higher vascular invasion risk in TCGA-LIHC dataset. E) Survival analysis was conducted using the data from TCGA-LIHC dataset. *p* < 0.001, HR = 2.24 (1.48-3.39) by log-rank test. F) Kaplan-Meier analysis of OS and DFS was performed according to the MCB1 levels in 160 HCC patients (cohort 1). Unless otherwise indicated, p-values were determined by unpaired student’s t test (two-tail) and *, **, ***, indicate p-val < 0.05, < 0.01, < 0.001, respectively.


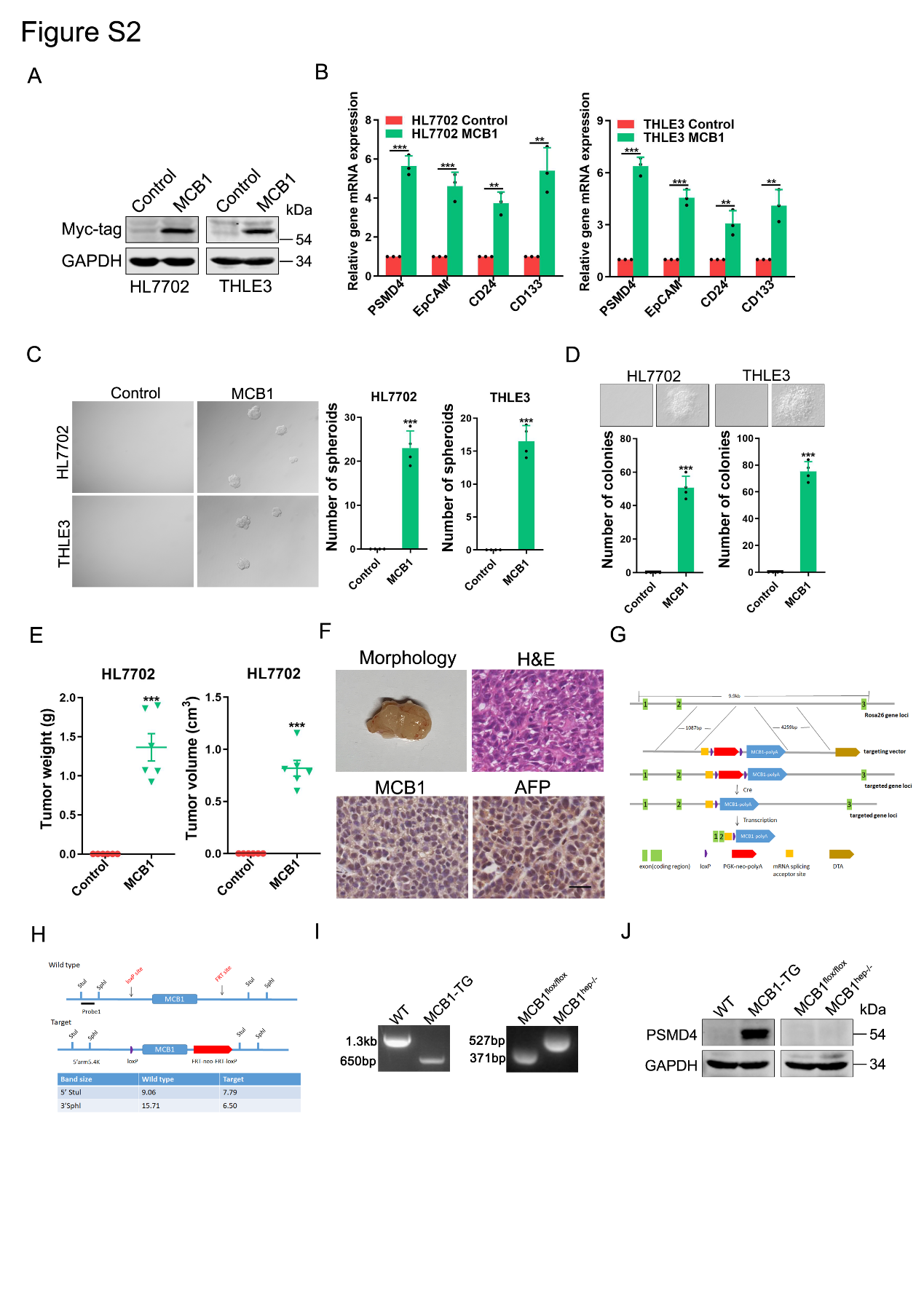


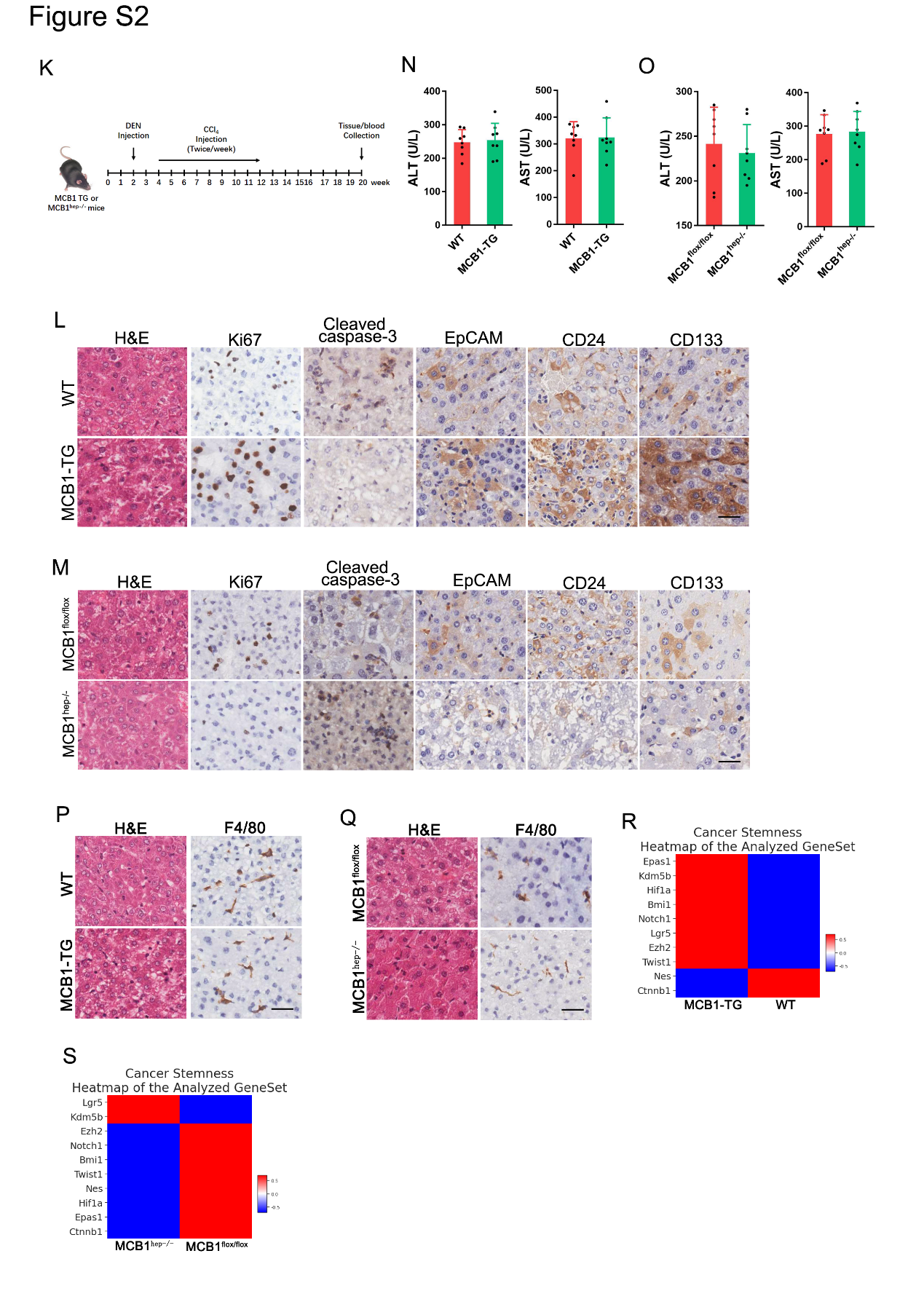


**Figure S2.** MCB1 drives HCC initiation. A) HL7702 and THLE3 cells were infected with MCB1 overexpression virus and determined by western-blot assay. B) MCB1 overexpression and control hepatocytes were subjected to real-time PCR assays (n = 3). Data are presented as mean ± SD. C) MCB1 overexpression and control hepatocytes were subjected to spheroid formation assays (n = 4). Data are presented as mean ± SD. D) MCB1-overexpressing and control hepatocytes were subjected to colony formation assays (n = 4). Data are presented as mean ± SD. E) HL7702 MCB1 and control cells were injected subcutaneously into NOD-SCID mice at 1×10^3^ cells per mouse. Tumor volume and tumor weight were measured 10 weeks later. Data are presented as mean ± SD. F) The tumor formed in E was subjected to H&E and IHC staining. Scale bar, 25 μm. G) Schematic of MCB1-TG mice. H) Schematic of MCB1^hep-/-^ mice. I) DNA was extracted from mice tails and amplified with primers as indicated. ROSA26^MCB1^; Alb-Cre-/- mice and MCB1^flox/flox^, Alb-Cre-/- mice were used as controls. J) Western-blot analysis of MCB1 protein in livers from MCB1-TG or MCB1^hep-/-^ and their WT control mice. K) HCC mouse model design. L) Representative images of H&E and IHC staining of Ki67, Caspase-3 and T-IC markers in liver tumors of the MCB1-TG and WT mice. Scale bar, 25 μm. M) Representative images of H&E and IHC staining of Ki67, Caspase-3 and T-IC markers in liver tumors of the MCB1^hep-/-^ and MCB1^flox/flox^ mice. Scale bar, 25 μm. N) The serum ALT and AST levels of MCB1-TG and WT mice at 5 months after DEN injection were examined (n = 8). Data are presented as mean ± SD. O) The serum ALT and AST levels of MCB1^hep-/-^ and MCB1^flox/flox^ mice at 5 months after DEN injection were examined (n = 8). Data are presented as mean ± SD. P) Representative images of H&E and IHC staining of F4/80 in liver tumors of MCB1-TG and WT mice. Scale bar, 25μm. Q) Representative images of H&E and IHC staining of F4/80 in liver tumors of MCB1^hep-/-^ and MCB1^flox/flox^ mice. Scale bar, 25μm. R) Heatmap showing the most differentially expressed stemness-associated genes in the HCCs from MCB1-TG mice at 5 months after DEN injection as compared with WT control mice. S) Heatmap showing the most differentially expressed stemness-associated genes in the HCCs from MCB1^hep-/-^ mice at 5 months post DEN injection as compared with WT control mice. Unless otherwise indicated, p-values were determined by unpaired student’s t test (two-tail) and **, ***, indicate p-val < 0.01, < 0.001, respectively.


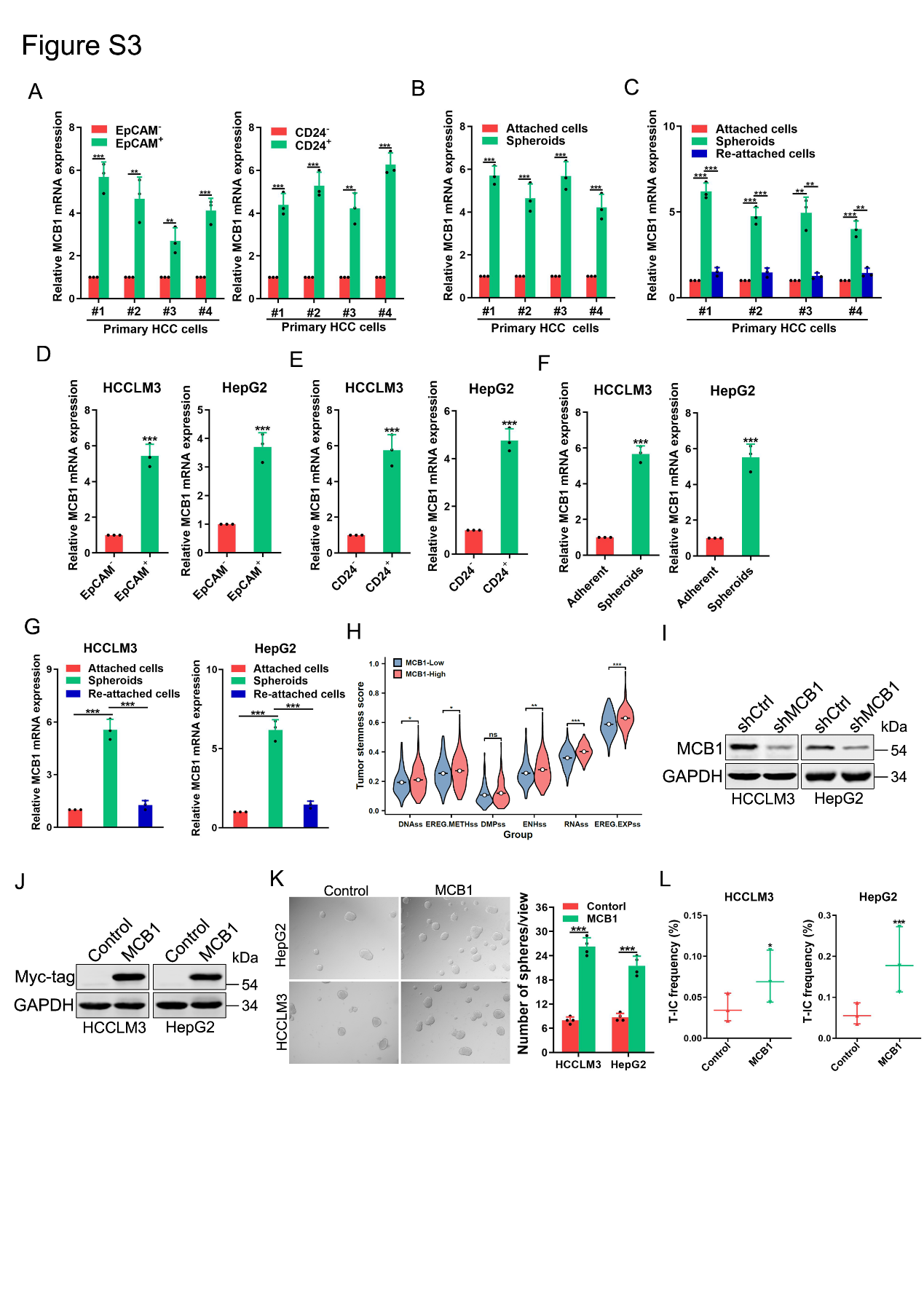


**Figure S3.** MCB1 facilitates liver T-ICs self-renewal. A) Real-time PCR analysis of MCB1 expression in sorted EpCAM^+^ or CD24^+^ primary HCC cells relative to negative cells (n = 3). Data are presented as mean ± SD. B) Real-time PCR analysis of MCB1 expression in primary HCC adherent cells and spheres (n = 3). Data are presented as mean ± SD. C) Real-time PCR analysis of MCB1 expression in primary adherent HCC cells, spheres and readherent cells (n = 3). Data are presented as mean ± SD. D) Real-time PCR analysis MCB1 expression in sorted EpCAM^+^ HCC cells relative to negative cells (n = 3). Data are presented as mean ± SD. E) Real-time PCR analysis MCB1 expression in sorted CD24^+^ HCC cells relative to negative cells (n = 3). F) Real-time PCR analysis of MCB1 expression in HCC adherent cells and spheres (n = 3). Data are presented as mean ± SD. G) Real-time PCR analysis of MCB1 expression in HCC adherent, spheres and re-adherent cells (n = 3). Data are presented as mean ± SD. H) Tumor stemness indices were calculated in different MCB1 expression groups. DNAss: DNA methylation-based stemness score, EREG-METHss: epigenetically regulated DNA methylation-based stemness score, DMPss: differentially methylated probe-based stemness score, ENHss: enhancer element stemness score, RNAss: RNA expression-based stemness score, EREG-EXPss: epigenetically regulated RNA expression-based stemness score. I) Western-blot analysis of MCB1 levels in MCB1 knockdown and control hepatoma cells. J) Western-blot analysis of MCB1 levels in MCB1 overexpression and control hepatoma cells. K) Representative images of hepatoma spheroids generated from MCB1 overexpression and control hepatoma cells. The number of spheroids was counted and compared (n = 4). Data are presented as mean ± SD. L) The frequency of liver T-ICs in MCB1 overexpression and control hepatoma cells was compared by *in vitro* limiting dilution assay. Unless otherwise indicated, p-values were determined by unpaired student’s t test (two-tail) and *, **, ***, NS indicate p-val <0.05, < 0.01, < 0.001, not significant respectively.


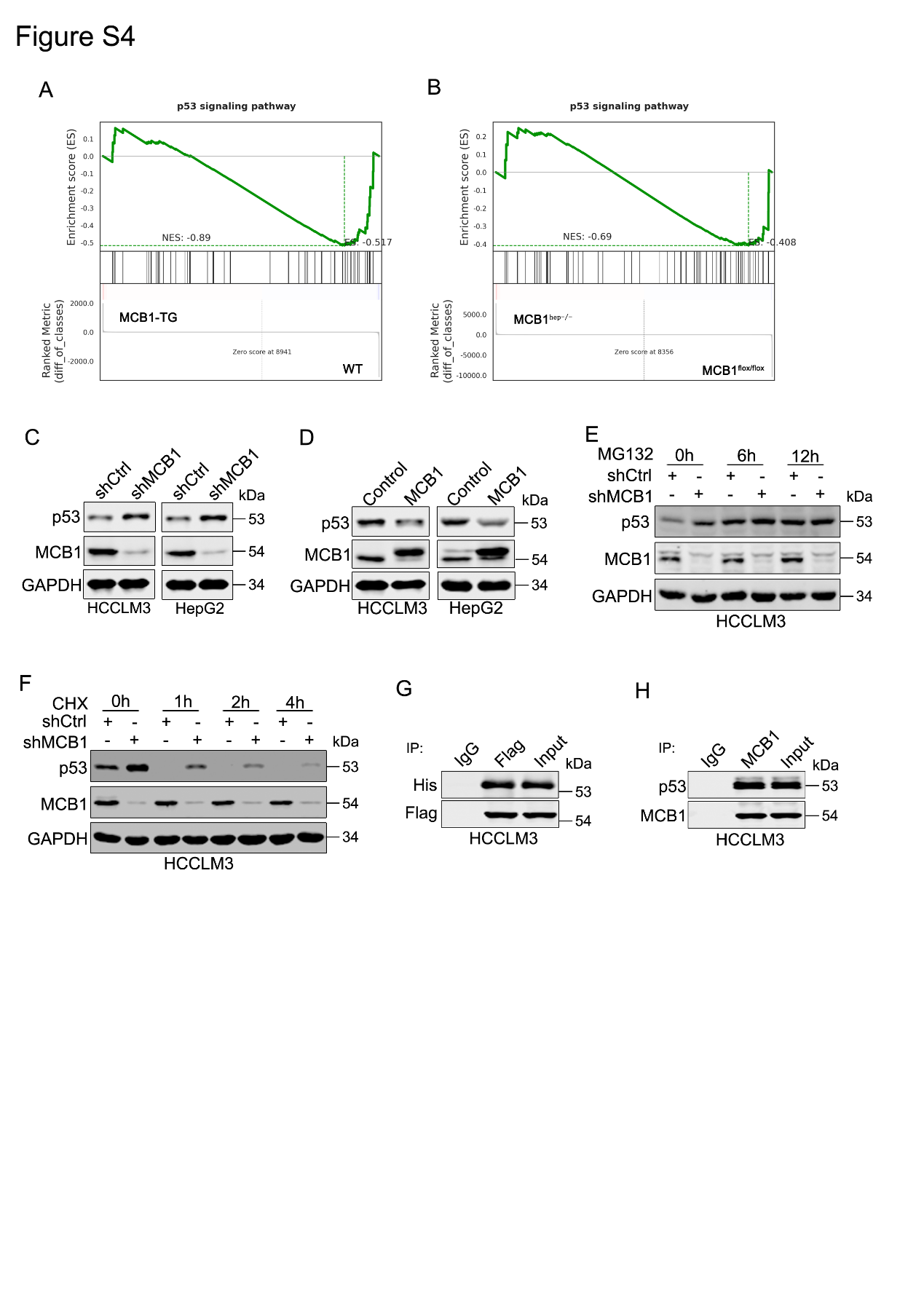


**Figure S4.** MCB1 decreases p53 expression in HCC cells. A) Gene set enrichment analysis showed the enrichment of gene sets negatively correlated with p53 pathway in DEN-induced HCCs from MCB1-TG mice compared with WT control mice. B) Gene set enrichment analysis shows the enrichment of gene sets negatively correlated with p53 pathway in DEN-induced HCCs from MCB1^hep-/-^ mice compared with WT control mice. C) Western blot analysis of p53 activation in the MCB1 knockdown hepatoma cells. D) Western-blot analysis of protein levels of p53 in MCB1 overexpression cells and control hepatoma cells. E) MCB1 knockdown cells or control hepatoma cells were treated with MG132 (20 μM) for the indicated times and then subjected to western blot analysis. F) MCB1 knockdown cells or control hepatoma cells were treated with CHX (100 μg/ml) for indicated times and then subjected to western-blot analysis. G) HCCLM3 cells were co-transfected with His-tagged P53 and Flag-tagged MCB1. Total cell lysates were subjected to immunoprecipitation with agarose conjugated anti-Flag and anti-IgG antibodies, and analyzed by immunoblotting with antibody against His and Flag. H) HCCLM3 cell lysates were subjected to immunoprecipitation with agarose conjugated anti-MCB1 and anti-IgG antibodies, and analyzed by immunoblotting with antibody against p53 and MCB1.


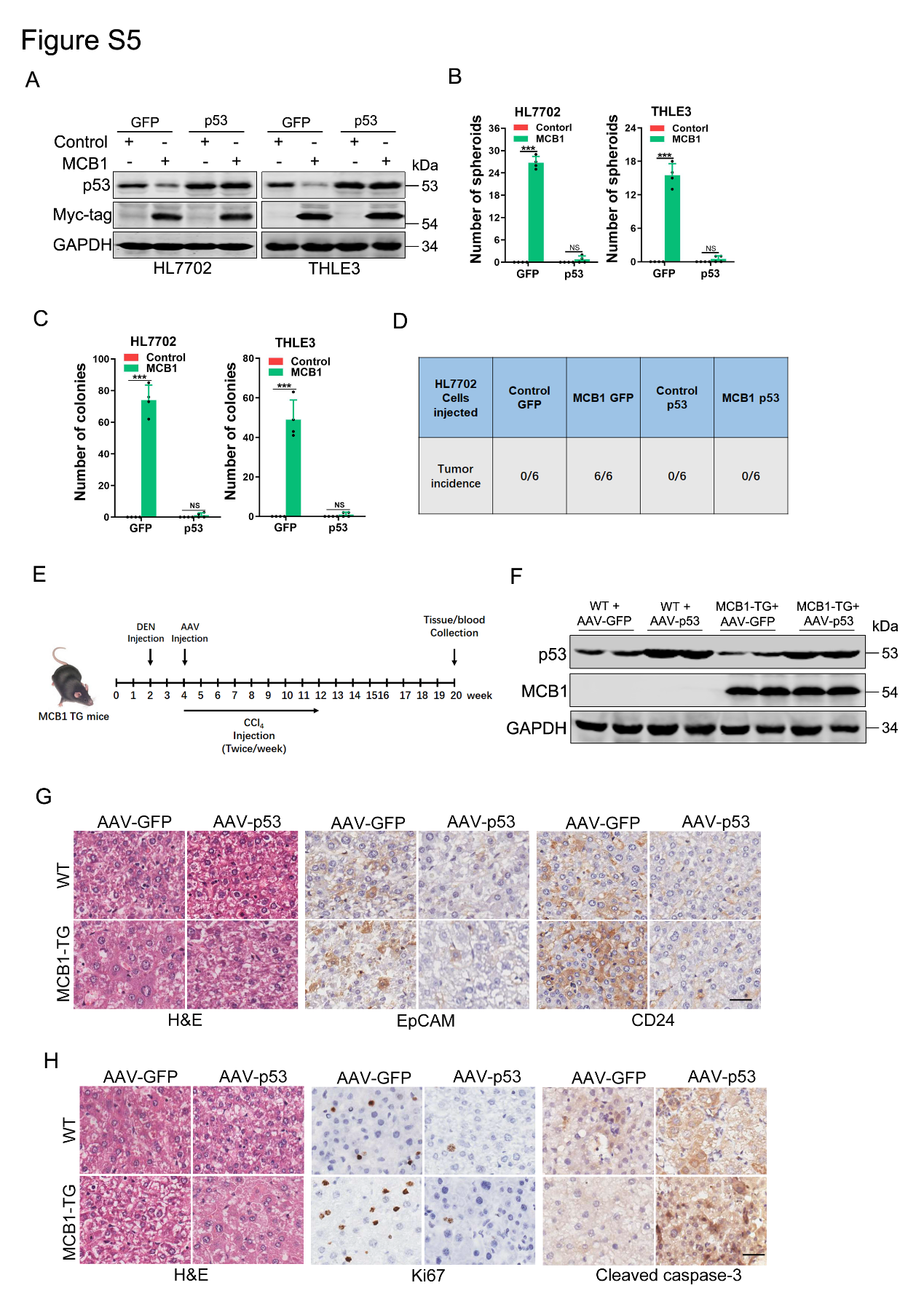

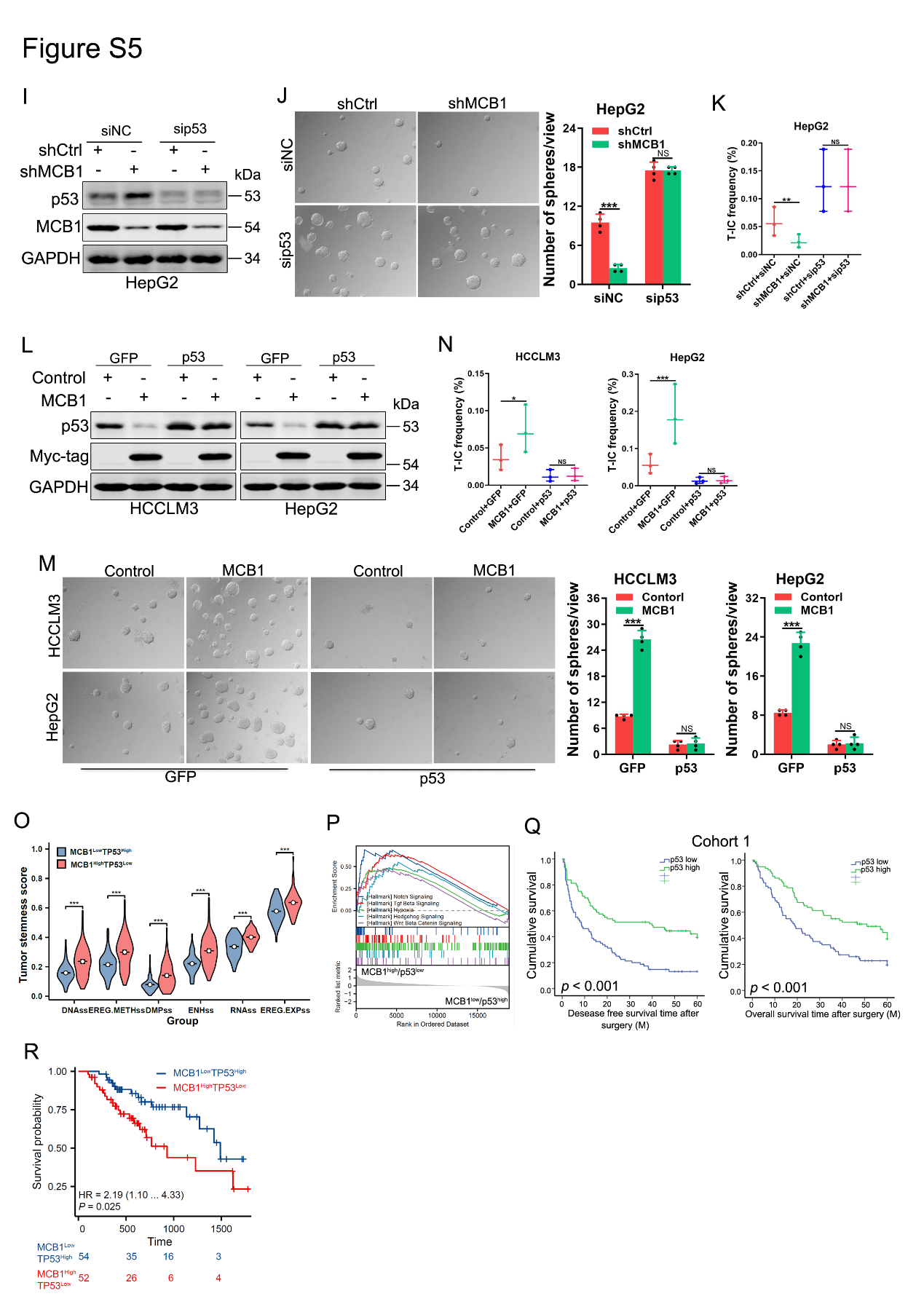


**Figure S5.** MCB1 promotes HCC development via targeting p53. A) HL7702 MCB1 cells and control cells were infected with p53 overexpression virus and were then subjected to western-blot assay. B) MCB1 overexpression hepatocyte cells and control cells were infected with p53 overexpression virus and were then subjected to spheroids formation assay (n = 4). Data are presented as mean ± SD. C) MCB1-overexpressing hepatocytes and control cells were infected with p53 overexpression virus and were then subjected to a colony formation assay (n = 4). Data are presented as mean ± SD. D) HL7702 MCB1 cells and control cells were infected with p53 overexpression virus and then injected subcutaneously into NOD-SCID mice at 1×10^3^ cells per mouse. Xenografted tumor formation was monitored 10 weeks later. E) Experimental design of HCC mouse model of p53 AAV overexpression. F) Western-blot analysis of p53 and MCB1 expression in MCB1-TG and WT mice. G) Representative images of H&E and IHC staining of T-IC markers in liver tumors of four groups mouse. Scale bar, 25μm. H) Representative images of H&E and IHC staining of Ki67 and Caspase-3 in liver tumors of four groups mouse. Scale bar, 25μm. I) MCB1 knockdown cells and control hepatoma cells was infected with sip53 or siNC and then subjected to western-blot assay. J, K) MCB1 knockdown and control hepatoma cells were transfected with sip53 or siNC and then subjected to spheroid formation assays (n = 4) Data are presented as mean ± SD. (J); *in vitro* limiting dilution assay (K). L) MCB1 overexpression cells and control hepatoma cells were infected with p53 overexpression virus or control virus and then subjected to western-blot assay. M) MCB1 overexpression cells and control hepatoma cells were infected with p53 overexpression virus or control virus and then subjected to spheroids formation assay (n = 4). Data are presented as mean ± SD. N) MCB1 overexpression cells and control hepatoma cells were infected with p53 overexpression virus or control virus and then subjected to *in vitro* limiting dilution assay. O) The tumor stemness scores were much higher in MCB1^High^ p53^low^ HCCs than those in MCB1^low^ p53^High^ HCCs. P) GSEA was conducted using TCGA-LIHC dataset to compare the stemness property between MCB1^High^ p53^Low^ HCCs and MCB1^low^ p53 ^High^ HCCs, and five vital stemness-related pathways were enriched. Q) OS and DFS was compared between the patients exhibiting low p53 and high p53 groups in cohort 1 using Kaplan-Meier analysis. R) Kaplan-Meier analysis was conducted to compare the prognosis between MCB1^High^p53^low^ patients and MCB1^low^ p53^High^ patients in TCGA-LIHC dataset. P = 0.025, HR = 2.19 (1.10-4.33) by log-rank test. Unless otherwise indicated, p-values were determined by unpaired student’s t test (two-tail) and *, **, ***, NS indicate p-val < 0.05, < 0.01, < 0.001, not significant respectively.


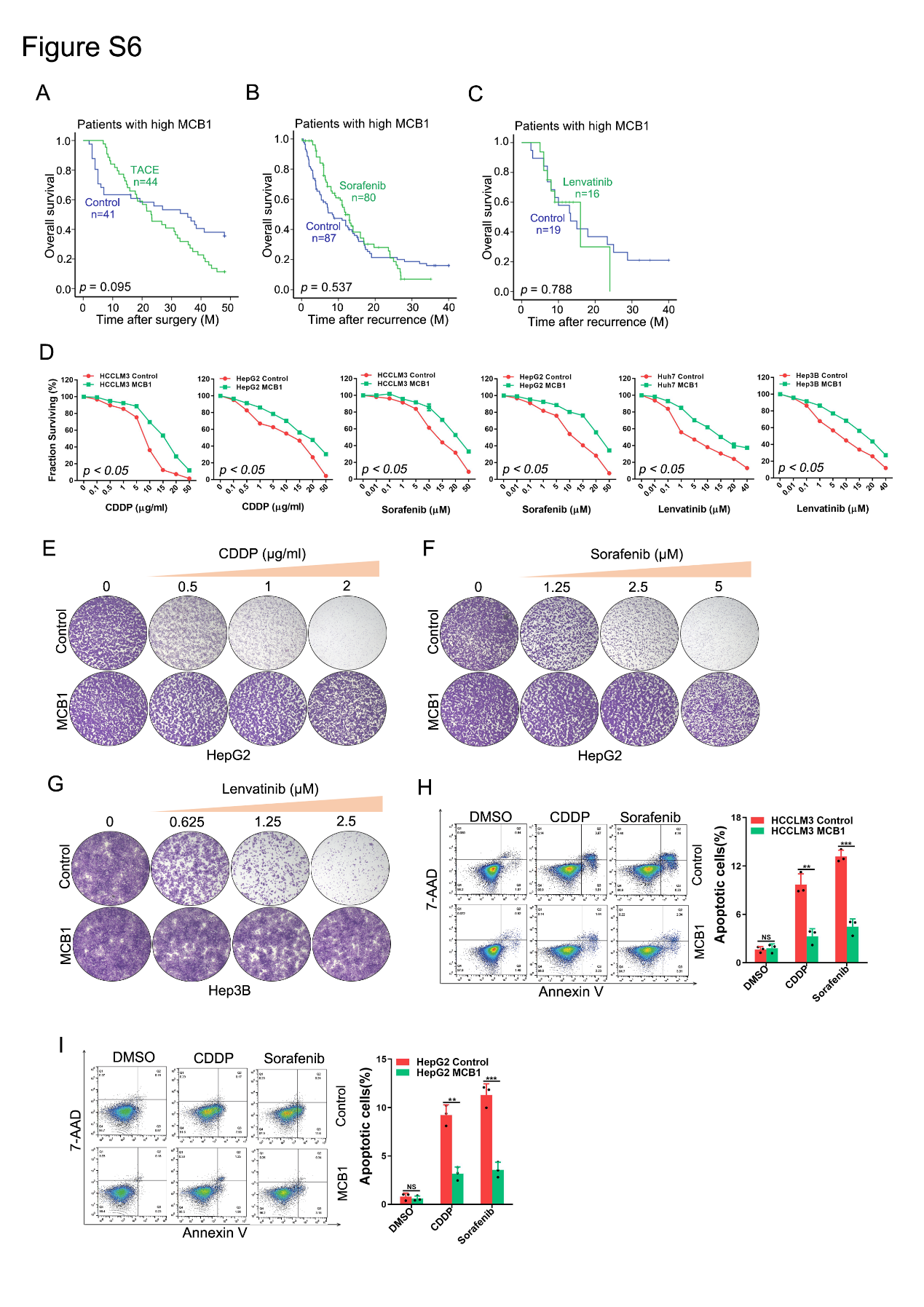


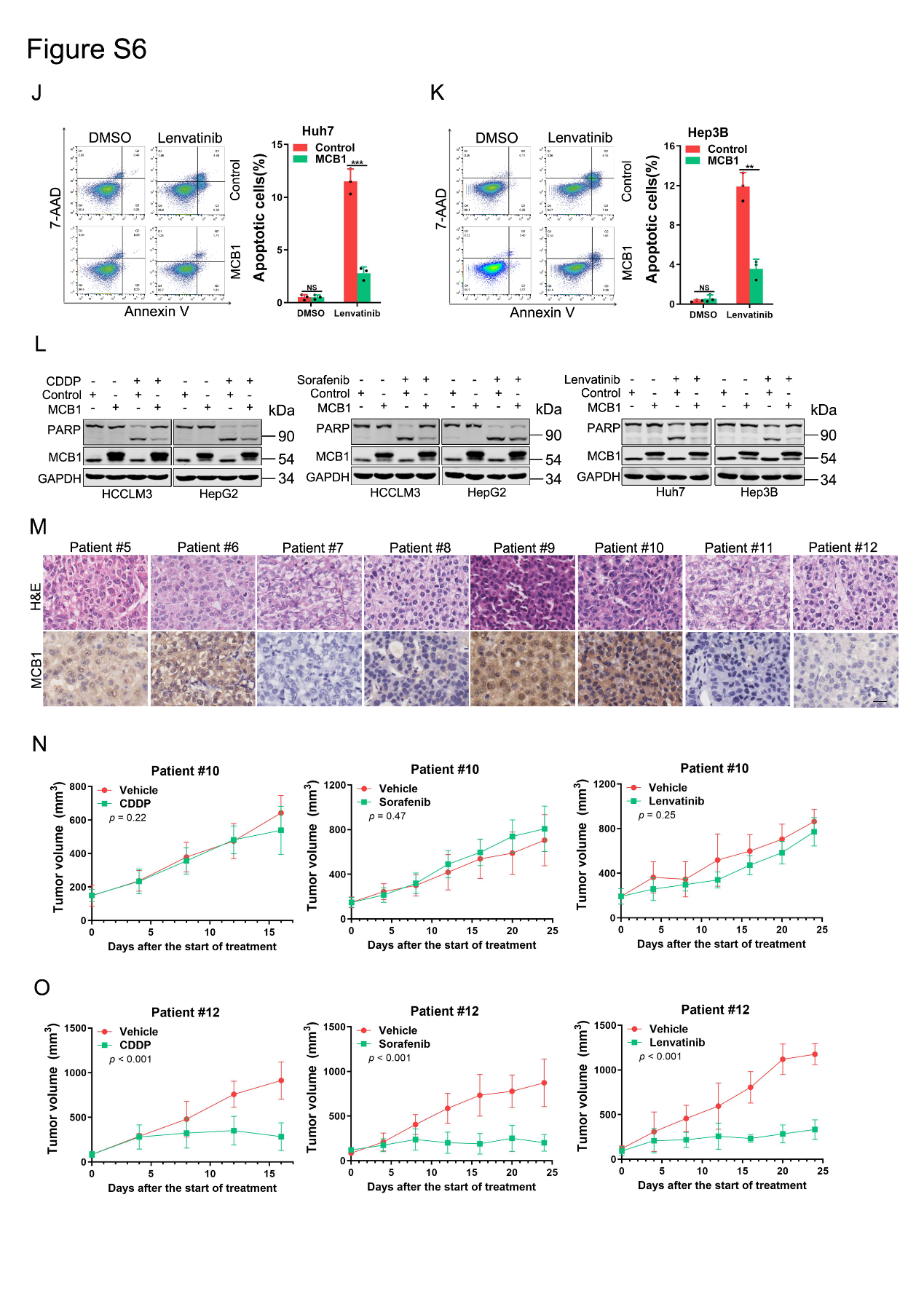

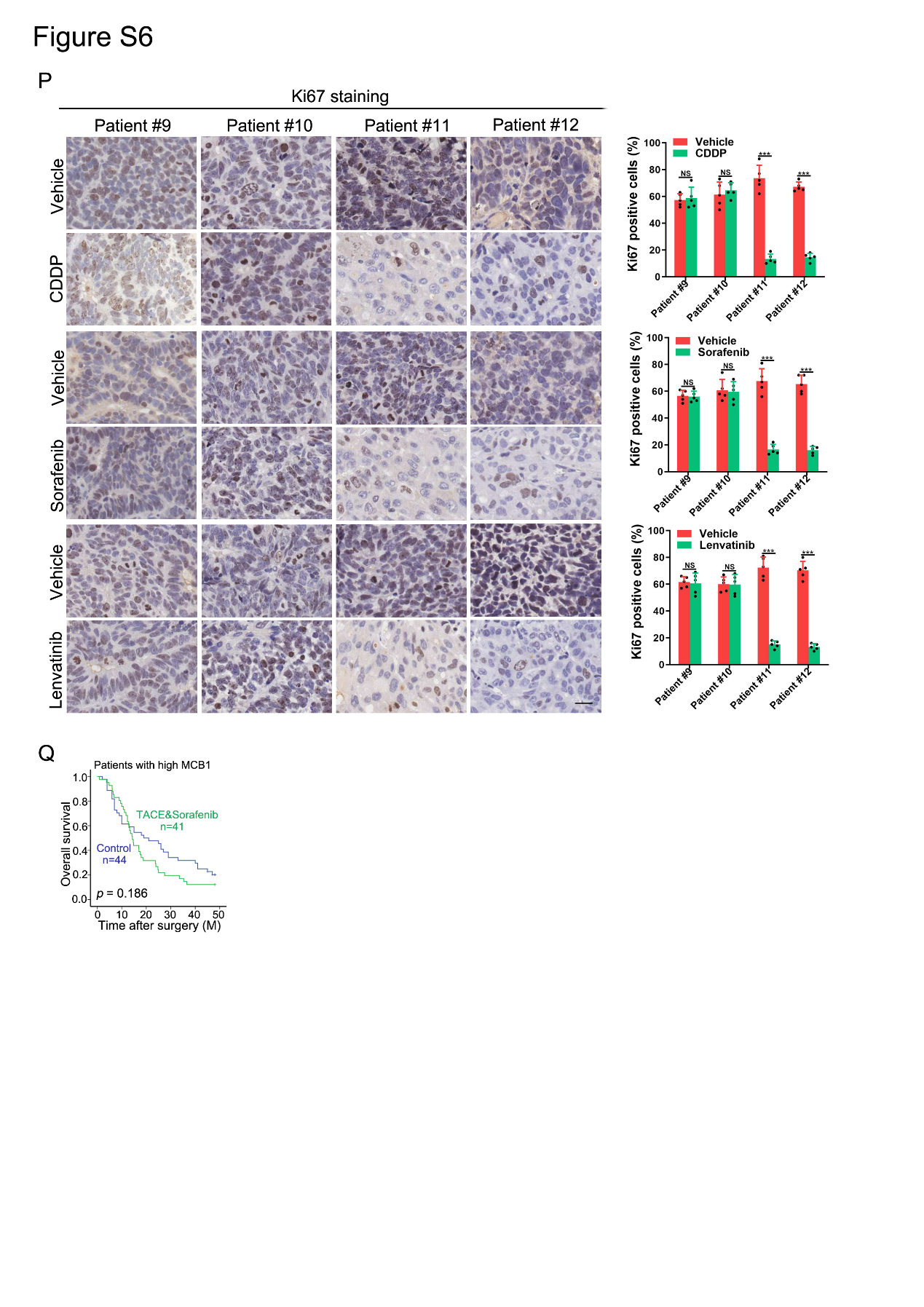


**Figure S6.** MCB1 determines the response of HCC cells upon CDDP and targeted drugs. A) Overall survival rates of HCC patients with high MCB1 levels treated with TACE (n = 44) or not (n = 41) after surgery were compared using Kaplan-Meier analysis (*p* = 0.095). B) Overall survival rates of HCC patients with high MCB1 levels treated with sorafenib (n = 80) or not (n = 87) after HCC relapse were compared using Kaplan-Meier analysis (*p* = 0.537). C) Overall survival rates of HCC patients with high MCB1 levels treated with lenvatinib (n = 16) or not (n = 19) after HCC relapse were compared using Kaplan-Meier analysis (*p* = 0.788). D) MCB1 overexpression and control hepatoma cells were treated with CDDP, sorafenib or lenvatinib for 48 hours and their cell survival curves was calculated. Data are presented as mean ± SD. E) HepG2 MCB1 and control cells were treated with CDDP for 10 days and their colony growth was examined. F) HepG2 MCB1 and control cells were treated with sorafenib for 10 days and their colony growth was examined. G) Hep3B MCB1 and control cells were treated with lenvatinib for 10 days and their colony growth was examined. H-K) MCB1-overexpressing and control hepatoma cells were treated with CDDP or targeted drugs for 48 hours, and apoptosis was examined by flow cytometry (n = 3). Data are presented as mean ± SD. L) MCB1-overexpressing and control hepatoma cells were treated with CDDP or targeted drugs for 48 hours and then subjected to western blot analysis. M) H&E and IHC staining of MCB1 in the primary HCCs. Scale bar, 25μm. N), O) PDXs derived from primary HCCs with high MCB1 levels (Patients #10) or low MCB1 levels (Patients #12) were treated with CDDP (2 g/kg body weight), sorafenib (30 mg/kg body weight), lenvatinib (4 mg/kg body weight) or vehicle (n = 5 for each group). Xenograft growth was monitored. Data are presented as mean ± SD. P) PDXs derived from indicated patients treated with CDDP or targeted drugs were subjected to Ki67 staining. Representative views were shown. Scale bar, 25μm. The proportion of the Ki67 positive cells was quantified (n = 5). Data are presented as mean ± SD. Q) Overall survival rates of HCC patients with high MCB1 levels received postsurgical TACE and sorafenib (n = 41) or not (n = 44) were compared using Kaplan-Meier analysis (*p* = 0.186). Unless otherwise indicated, p-values were determined by unpaired student’s t test (two-tail) and *, **, ***, NS indicate p-val < 0.05, < 0.01, < 0.001, not significant respectively.


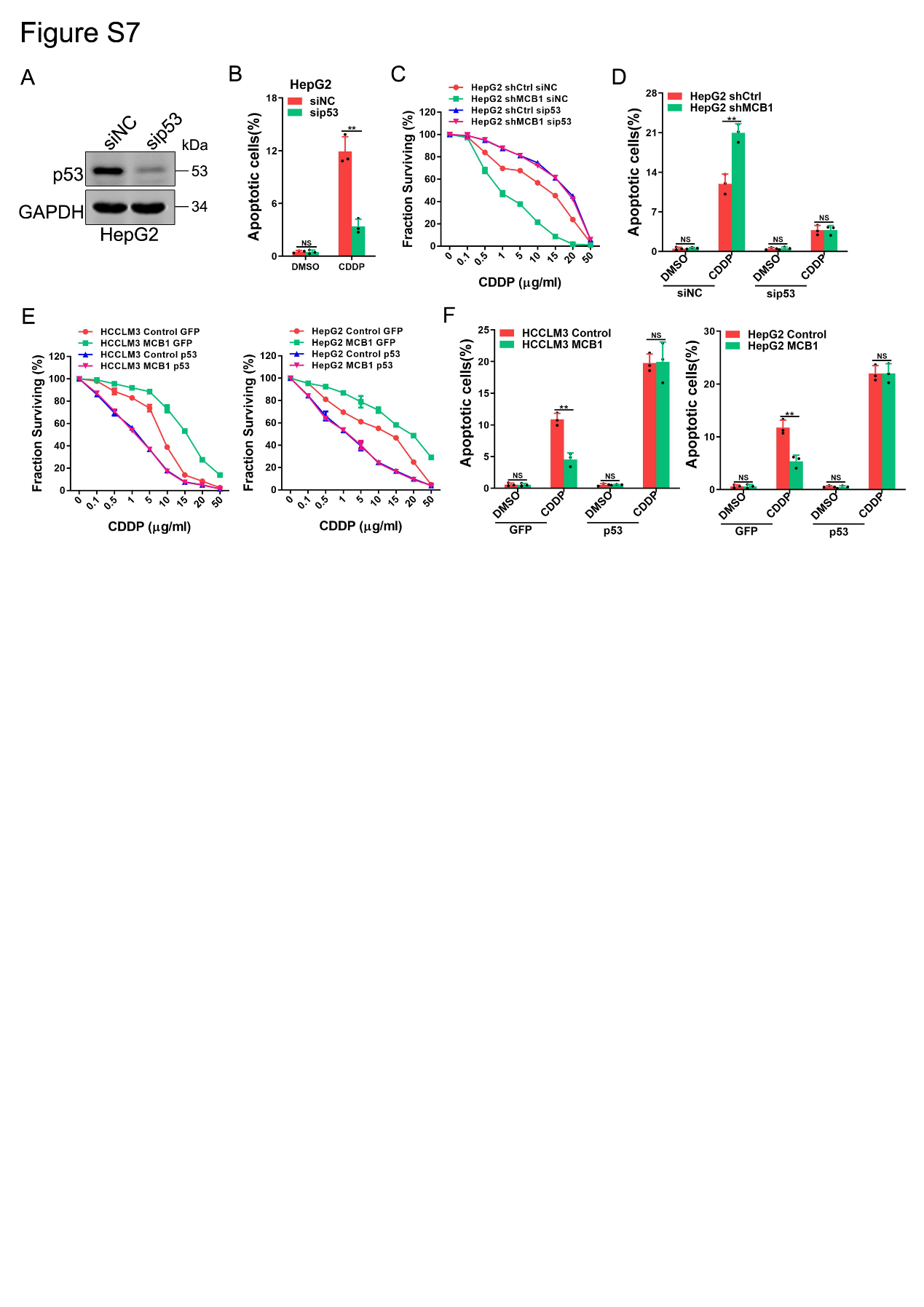


**Figure S7.** MCB1 mediated chemo-drugs resistance via degradation of p53. A) Hepatoma cells were transfected with sip53 or siNC for 48 hours and then subjected to western-blot analysis. B) Hepatoma cells transfected with sip53 or siNC were treated with CDDP (4 µg/ml) for 48 hours followed by cytometry analysis of apoptosis (n = 3). Data are presented as mean ± SD.C) MCB1 knockdown cells and control hepatoma cells was infected with sip53 or siNC. The cells were then treated with CDDP for 48 hours and cell survival curves was calculated. Data are presented as mean ± SD. D) MCB1 knockdown cells and control hepatoma cells was infected with sip53 or siNC. The cells were then treated with CDDP for 48 hours and their apoptosis was examined by flow cytometry (n = 3). Data are presented as mean ± SD. E) MCB1 overexpression cells and control hepatoma cells were infected with p53 overexpression virus or control virus. The cells were then treated with CDDP for 48 hours and cell survival curves was calculated. Data are presented as mean ± SD. F) MCB1 overexpression cells and control hepatoma cells were infected with p53 overexpression virus or control virus. The cells were then treated with CDDP for 48 hours and their apoptosis was examined by flow cytometry (n = 3). Data are presented as mean ± SD. Unless otherwise indicated, p-values were determined by unpaired student’s t test (two-tail) and **, NS indicate p-val < 0.01, not significant respectively.


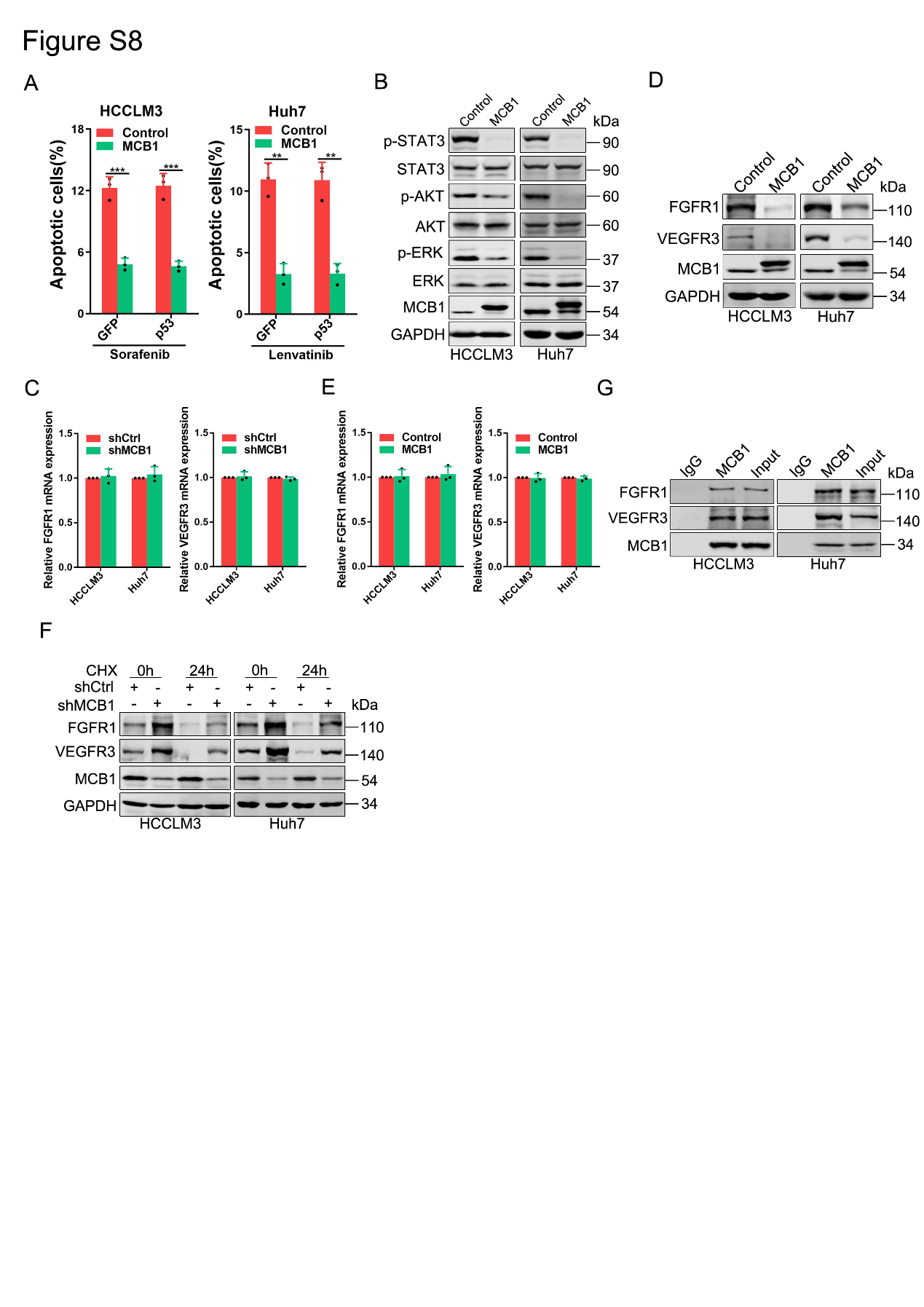


**Figure S8.** MCB1 downregulates FGFR1 and VEGFR3 expression via proteasome-dependent degradation. A) MCB1 knockdown cells and control hepatoma cells was infected with sip53 or siNC. The cells were then treated with sorafenib or lenvatinib for 48 hours and their apoptosis was examined by flow cytometry (n = 3). Data are presented as mean ± SD. B) Western-blot analysis of phosphorylation of STAT3, AKT and ERK in MCB1 overexpression cells and control hepatoma cells. C) The mRNA levels of FGFR1 and VEGFR3 in MCB1 knockdown cells and control hepatoma cells were examined by real-time PCR analysis (n = 3). Data are presented as mean ± SD. D) The protein levels of FGFR1 and VEGFR3 in MCB1 overexpression cells and control hepatoma cells were examined by western-blot analysis. E) The mRNA levels of FGFR1 and VEGFR3 in MCB1 overexpression cells and control hepatoma cells were examined by real-time PCR analysis (n = 3). Data are presented as mean ± SD. F) MCB1 knockdown cells or control hepatoma cells were treated with CHX for the indicated times and then subjected to western blot analysis. G) Endogenous FGFR1, VEGFR3 and MCB1 were immunoprecipitated. Data are presented as mean ± SD. Unless otherwise indicated, p-values were determined by unpaired student’s t test (two-tail) and **, *** indicate p-val < 0.01, <0.001 respectively.


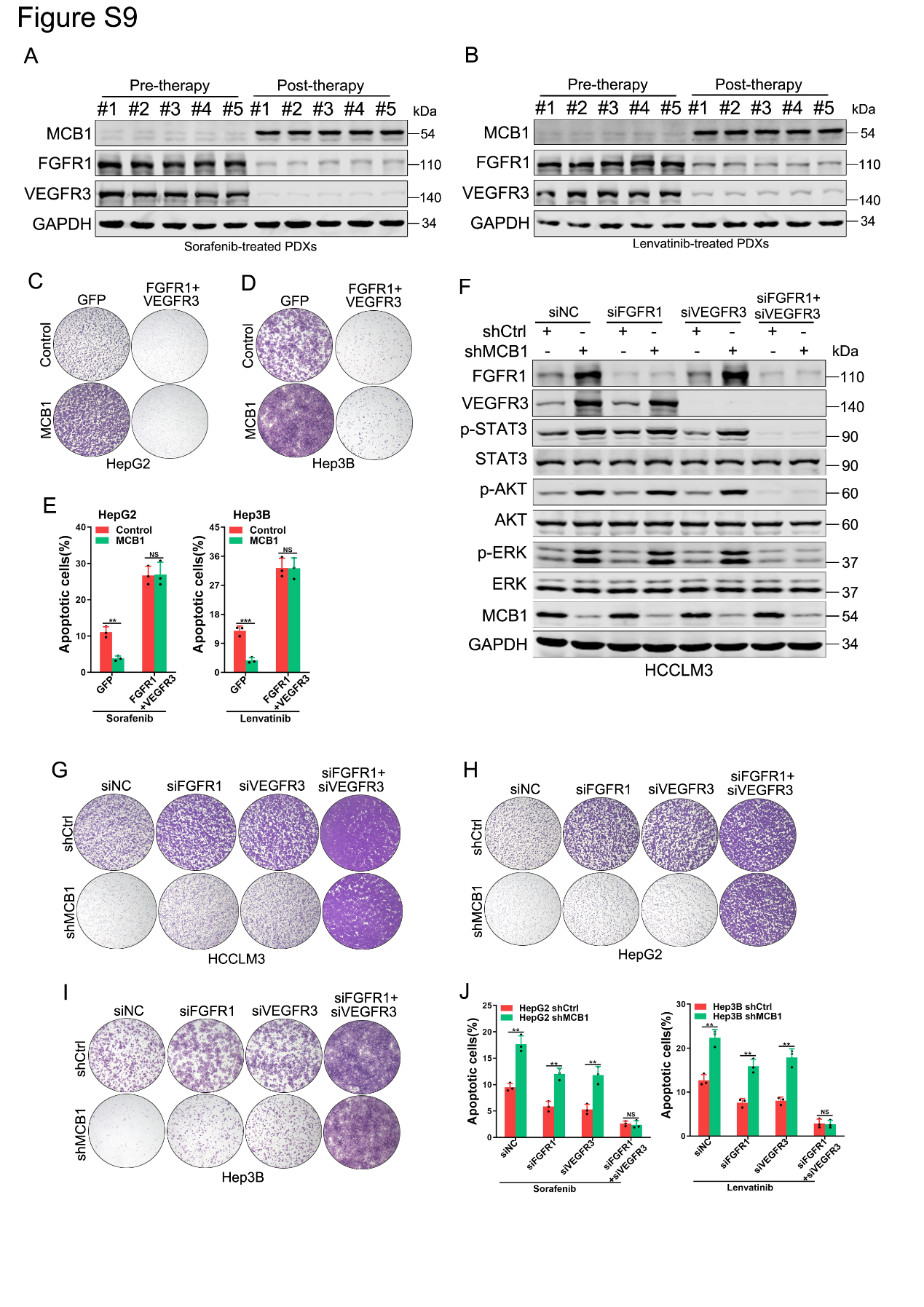


**Figure S9.** MCB1 mediated targeted drugs resistance via degradation of TKI targets. A) Western blot analysis of indicated proteins in sorafenib pre-therapy and post-therapy PDXs. B) Western blot analysis of indicated proteins in lenvatinib pre-therapy and post-therapy PDXs. C) Colony formation assay of HepG2 MCB1 and control cells infected with lentivirus expressing FGFR1/VEGFR3 upon sorafenib treatment (1.25 μM) in a 12-well dish for 10 days. D) Colony formation assay of Hep3B MCB1 and control cells infected with lentivirus expressing FGFR1/VEGFR3 upon lenvatinib treatment (0.625 μM) in a 12-well dish for 10 days. E) MCB1 overexpression cells and control hepatoma cells infected with lentivirus expressing FGFR1/VEGFR3 were treated with targeted drugs for 48 hours followed by cytometry analysis of apoptosis (n = 3). Data are presented as mean ± SD. F) MCB1 knockdown cells and control hepatoma cells were transfected with siFGFR1, siVEGFR3 or siNC for 48 hours and then subjected to western-blot analysis. G) Colony formation assay of HCCLM3 shMCB1 and control cells transfected with siFGFR1, siVEGFR3 or siNC with sorafenib treatment (1.25 μM) in a 12-well dish for 10 days. H) Colony formation assay of HepG2 shMCB1 and control cells transfected with siFGFR1, siVEGFR3 or siNC with sorafenib treatment (1.25 μM) in a 12-well dish for 10 days. I) Colony formation assay of Hep3B shMCB1 and control cells transfected with siFGFR1, siVEGFR3 or siNC with lenvatinib treatment (0.625 μM) in a 12-well dish for 10 days. J) MCB1 knockdown cells and control hepatoma cells transfected with siFGFR1, siVEGFR3 or siNC were treated with targeted drugs for 48 hours followed by cytometry analysis of apoptosis (n = 3). Data are presented as mean ± SD. Unless otherwise indicated, p-values were determined by unpaired student’s t test (two-tail) and **, ***, NS indicate p-val < 0.01, < 0.001, not significant, respectively.


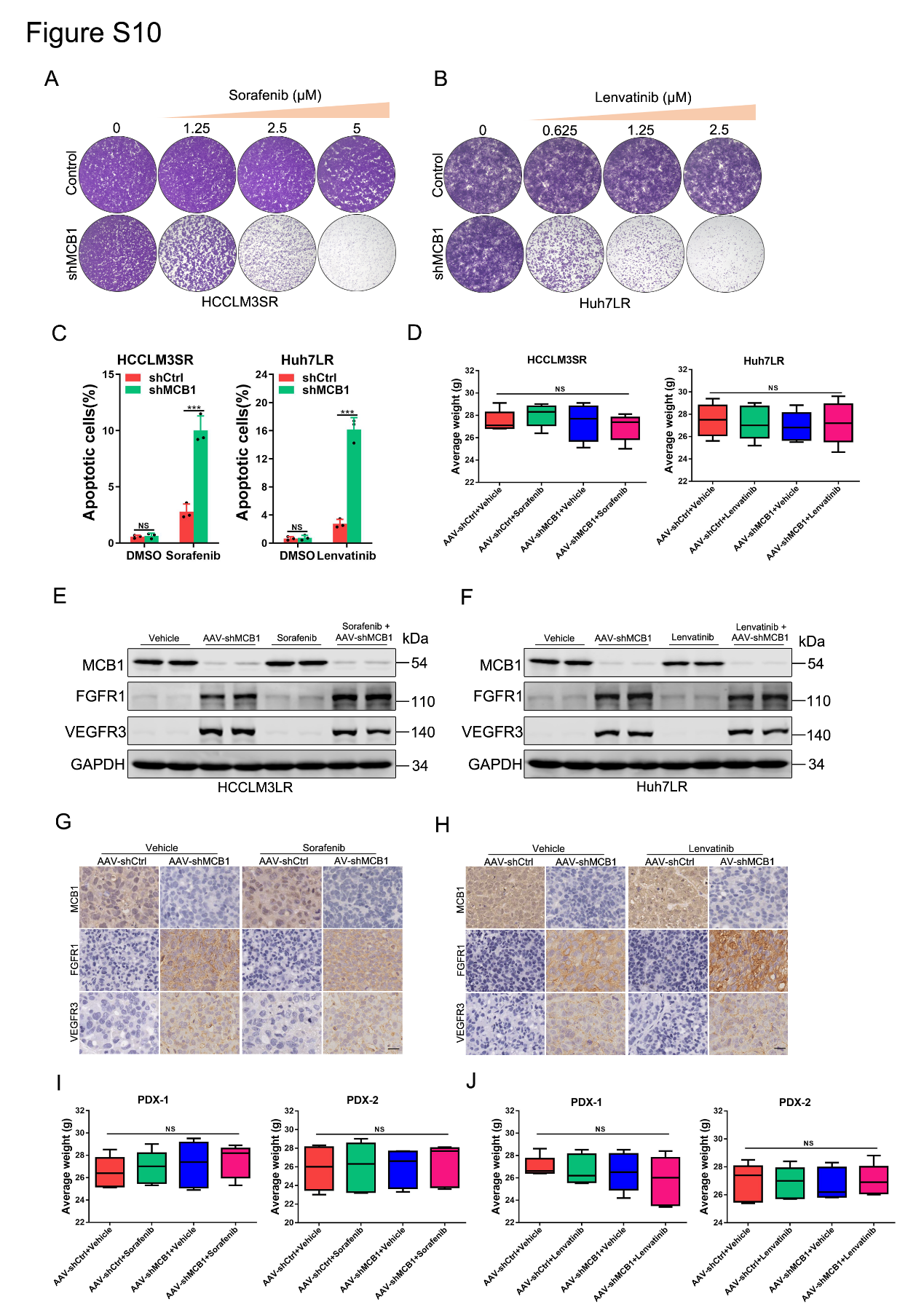

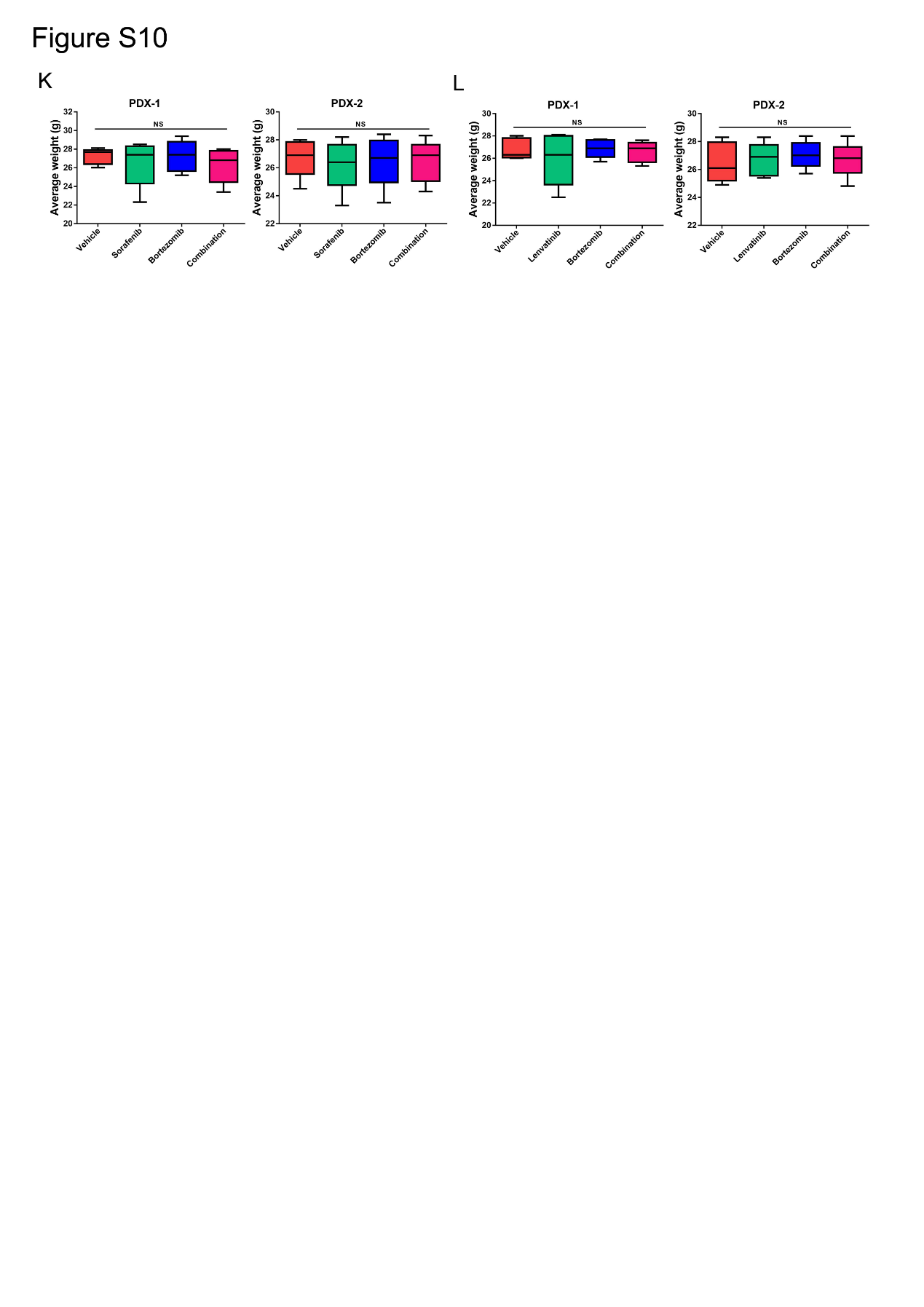


**Figure S10.** Targeting MCB1 restores the response of targeted drugs in HCC. A) HCCLM3SR shMCB1 and control cells treated with sorafenib for 10 days and their colony growth was examined. B) HCCLM3SR shMCB1 and control cells treated with lenvatinib for 10 days and their colony growth was examined. C) HCCLM3SR/Huh7LR shMCB1 and control cells were treated with targeted drugs or 48 hours and their apoptosis was examined by flow cytometry (n = 3). Data are presented as mean ± SD. D) Average weight of HCCLM3SR-luc or Huh7R-luc cells orthotopically xenografted mice (n = 8 per group) after indicated treatments. The horizontal lines in the box plots represent the medians, the boxes represent the interquartile range, and the whiskers represent the minimum and maximum values. Data are presented as mean ± SD. E) Western blot analysis of indicated proteins in HCCLM3SR-luc xenografts after indicated treatments. F) Western blot analysis of indicated proteins in Huh7R-luc xenografts after indicated treatments. G), H) IHC staining of MCB1, FGFR1 and VEGFR3 in PDXs after indicated treatments. Scale bar, 25μm. I)-L) Average weight of mice (n = 5 per group) xenografted with PDXs after indicated treatments. The horizontal lines in the box plots represent the medians, the boxes represent the interquartile range, and the whiskers represent the minimum and maximum values. Unless otherwise indicated, p-values were determined by unpaired student’s t test (two-tail) and ***, NS indicate p-val < 0.001, not significant, respectively.

**Supplementary tables**

**Table S1. Four candidate genes**

| KRTCAP2 | MCB1 | TMCO1 | ADAM15 |
| --- | --- | --- | --- |

**Table S2. Clinicopathologic features of 160 HCC specimens in cohort 1.**

| Characteristics |  |  | MCB1 low (n=80) | MCB1 high (n=80) | p value | p53 low (n=80) | p53 high (n=80) | p value |
| --- | --- | --- | --- | --- | --- | --- | --- | --- |
| Age(year) | ≤50 | | 43 | 47 | p>0.05 | 40 | 50 | p>0.05 |
|  | >50 | | 37 | 33 |  | 40 | 30 |  |
| Gender | Male | | 65 | 68 | p>0.05 | 67 | 66 | p>0.05 |
|  | Female | | 15 | 12 |  | 13 | 14 |  |
| HBsAg | Positive | | 59 | 73 | p<0.05 | 68 | 64 | p>0.05 |
|  | Negative | | 21 | 7 |  | 12 | 16 |  |
| AFP(ng/mL) | ≤400 | | 46 | 38 | p>0.05 | 39 | 45 | p>0.05 |
|  | >400 | | 34 | 42 |  | 41 | 35 |  |
| Tumor size(cm) | ≤5 | | 32 | 30 | p>0.05 | 24 | 38 | p<0.05 |
|  | >5 | | 48 | 50 |  | 56 | 42 |  |
| Tumor number | Single | | 65 | 62 | p>0.05 | 62 | 65 | p>0.05 |
|  | Multiple | | 15 | 18 |  | 18 | 15 |  |
| Portal vein tumor thrombus | Yes | | 8 | 13 | p>0.05 | 9 | 12 | p>0.05 |
|  | No | | 72 | 67 |  | 71 | 68 |  |
| Microvascular invasion | Yes | | 28 | 45 | p<0.05 | 39 | 34 | p>0.05 |
|  | No | | 52 | 35 |  | 41 | 46 |  |
| Encapsulation | Complete | | 32 | 21 | p>0.05 | 26 | 27 | p>0.05 |
|  | None | | 48 | 59 |  | 54 | 53 |  |
| Pathologic satellite | Yes | | 32 | 49 | p<0.05 | 43 | 38 | p>0.05 |
|  | No | | 48 | 31 |  | 37 | 42 |  |
| BCLC stage | 0-A | | 57 | 57 | p>0.05 | 59 | 55 | p>0.05 |
|  | B or C | | 23 | 23 |  | 21 | 25 |  |
| TNM | I-II | | 66 | 56 | p>0.05 | 60 | 62 | p>0.05 |
|  | III-IV | | 14 | 24 |  | 20 | 18 |  |

HBsAg, hepatitis B virus surface antigen; AFP, α-fetoprotein; TNM, Tumor-Nodes-Metastasis; BCLC, Barcelona Clinic Liver Cancer Staging.

**Table S3. Clinicopathologic features of 88 HCC specimens in cohort 2.**

| Characteristics |  | n=88 |
| --- | --- | --- |
| Age(year) | ≤50 | 40 |
|  | >50 | 48 |
| Gender | Male | 74 |
|  | Female | 14 |
| HBsAg | Positive | 82 |
|  | Negative | 6 |
| AFP(ng/mL) | ≤400 | 56 |
|  | >400 | 32 |
| Tumor size(cm) | ≤5 | 42 |
|  | >5 | 46 |
| Tumor number | Single | 68 |
|  | Multiple | 20 |
| Portal vein tumor thrombus | Yes | 17 |
|  | No | 71 |
| Microvascular invasion | Yes | 43 |
|  | No | 45 |
| Encapsulation | Complete | 42 |
|  | None | 46 |
| Pathologic satellite | Yes | 32 |
|  | No | 56 |
| BCLC stage | 0-A | 45 |
|  | B or C | 43 |
| TNM | I-II | 66 |
|  | III-IV | 22 |

HBsAg, hepatitis B virus surface antigen; AFP, α-fetoprotein; TNM, Tumor-Nodes-Metastasis; BCLC, Barcelona Clinic Liver Cancer Staging.

**Table S4. Clinicopathologic features of 159 HCC specimens in cohort 3.**

| Characteristics |  | n=159 |
| --- | --- | --- |
| Age(year) | ≤50 | 101 |
|  | >50 | 58 |
| Gender | Male | 151 |
|  | Female | 8 |
| HBsAg | Positive | 150 |
|  | Negative | 9 |
| AFP(ng/mL) | ≤400 | 90 |
|  | >400 | 69 |
| Tumor size(cm) | ≤5 | 31 |
|  | >5 | 128 |
| Portal vein tumor thrombus | Yes | 60 |
|  | No | 99 |
| BCLC stage | 0-A | 46 |
|  | B or C | 113 |
| TNM | I-II | 73 |
|  | III-IV | 86 |

HBsAg, hepatitis B virus surface antigen; AFP, α-fetoprotein; TNM, Tumor-Nodes-Metastasis; BCLC, Barcelona Clinic Liver Cancer Staging.

**Table S5. Clinicopathologic features of 31 HCC specimens in cohort 4.**

| Characteristics |  | n=31 |
| --- | --- | --- |
| Age(year) | ≤50 | 11 |
|  | >50 | 20 |
| Gender | Male | 22 |
|  | Female | 9 |
| HBsAg | Positive | 28 |
|  | Negative | 3 |
| AFP(ng/mL) | ≤400 | 19 |
|  | >400 | 12 |
| Tumor size(cm) | ≤5 | 15 |
|  | >5 | 16 |
| Tumor number | Single | 19 |
|  | Multiple | 12 |
| Portal vein tumor thrombus | Yes | 0 |
|  | No | 31 |
| Encapsulation | Complete | 6 |
|  | None | 25 |
| Pathologic satellite | Yes | 6 |
|  | No | 25 |
| BCLC stage | 0-A | 24 |
|  | B or C | 7 |
| TNM | I-II | 24 |
|  | III-IV | 7 |

HBsAg, hepatitis B virus surface antigen; AFP, α-fetoprotein; TNM, Tumor-Nodes-Metastasis; BCLC, Barcelona Clinic Liver Cancer Staging.

**Table S6. Clinicopathologic features of 83 HCC specimens in cohort 5.**

| Characteristics |  | n=83 |
| --- | --- | --- |
| Age(year) | ≤50 | 39 |
|  | >50 | 44 |
| Gender | Male | 74 |
|  | Female | 9 |
| HBsAg | Positive | 46 |
|  | Negative | 37 |
| AFP(ng/mL) | ≤400 | 41 |
|  | >400 | 42 |
| Tumor size(cm) | ≤5 | 25 |
|  | >5 | 58 |
| Tumor number | Single | 59 |
|  | Multiple | 24 |
| Portal vein tumor thrombus | Yes | 0 |
|  | No | 83 |
| Encapsulation | Complete | 30 |
|  | None | 53 |
| Pathologic satellite | Yes | 11 |
|  | No | 72 |
| BCLC stage | A | 51 |
|  | B or C | 32 |
| TNM | I-II | 61 |
|  | III-IV | 22 |

HBsAg, hepatitis B virus surface antigen; AFP, α-fetoprotein; TNM, Tumor-Nodes-Metastasis; BCLC, Barcelona Clinic Liver Cancer Staging.

**Table S7. Thirty-eight upregulated protein kinases upon MCB1 silencing.**

| EphB1 | EphA3 | PDGFR-α | Itk | TNK1 |
| --- | --- | --- | --- | --- |
| SRMS | Csk | Fyn | ROR1 | Btk |
| Hck | JAK2 | SYK | PYK2 | VEGFR3 |
| ROS | ALK | TRKB | RET | FRK |
| ABL1 | EphA5 | ZAP70 | Axl | FER |
| BMX | EGFR | RYK | MATK | EphA8 |
| JAK1 | EphA6 | TXK | EphA7 | Tyk2 |
| EphA4 | FGFR1 | Blk |  |  |

**Table S8. Fifty-four downregulated protein kinases upon MCB1 overexpressing.**

| FGFR1 | NGFR | ROR2 | LCK | Tie-1 |
| --- | --- | --- | --- | --- |
| SCFR | Tyk2 | M-CSFR | Lyn | Dtk |
| EphA1 | RET | EphA4 | EphA2 | PDGFR-β |
| MATK | HGFR | EphA6 | FRK | MUSK |
| FER | IGF-I R | ALK | Btk | LTK |
| JAK3 | FGFR2 | VEGFR2 | PDGFR-α | RYK |
| Tie-2 | ABL1 | EphA3 | JAK1 | EphA5 |
| Axl | EphB6 | FAK | BMX | PYK2 |
| ROS | ErbB3 | EphA8 | TXK | Itk |
| Fyn | EphA7 | SYK | ROR1 | Blk |
| EGFR | EphB1 | VEGFR3 | Csk |  |

**Table S9. Antibody list.**

| **Antigens** | **Manufacturer** | **Application** |
| --- | --- | --- |
| MCB1 | Proteintech Group, China | 1:1000 for WB or 1:100 for IHC |
| MCB1 | The International Cooperation Laboratory on Signal Transduction, EHBH, SMMU, China | Elisa |
| p53 | Proteintech Group, China | 1:1000 for WB or 1:100 for IHC or immunofluorescence staining (IF) |
| Ki67 | Proteintech Group, China | 1:100 for IHC |
| Caspase-3 | Cell Signaling Technology, Beverly, MA | 1:50 for IHC |
| p-STAT3 | Abcam, Cambridge, MA | 1:1000 for WB |
| STAT3 | Proteintech Group, China | 1:1000 for WB |
| p-AKT | Cell Signaling Technology, Beverly, MA | 1:1000 for WB |
| AKT | Proteintech Group, China | 1:1000 for WB |
| p-ERK | Abclonal, China | 1:1000 for WB |
| ERK | Cell Signaling Technology, Beverly, MA | 1:1000 for WB |
| VEGFR3 | Abcam, Cambridge, MA | 1:1000 for WB or 1:100 for IHC or immunofluorescence staining (IF) |
| AFP | Proteintech Group, China | 1:100 for IHC |
| FGFR1 | Proteintech Group, China | 1:1000 for WB or 1:100 for IHC or immunofluorescence staining (IF) |
| RET | Cell Signaling Technology, Beverly, MA | 1:1000 for WB |
| His | Proteintech Group, China | 1:1000 for WB or 1:50 for IP |
| Flag | Abcam, Cambridge, MA | 1:1000 for WB or 1:50 for IP |
| myc-tag | Proteintech Group, China | 1:1000 for WB |
| GST-tag | Proteintech Group, China | 1:1000 for WB |
| HA | Proteintech Group, China | 1:1000 for WB |
| PARP | Cell Signaling Technology, Beverly, MA | 1:1000 for WB |
| β-actin | Santa Cruz Biotechnology, CA | 1:2000 for WB |
| GAPDH | Santa Cruz Biotechnology, CA | 1:5000 for WB |
| EpCAM | Abcam, Cambridge, MA | 1:100 for IHC |
| EpCAM | Biolegend, Inc., San Diego, CA | 1:50 for FACS |
| CD24 | Santa Cruz Biotechnology, CA | 1:100 for IHC |
| CD24 | Biolegend, Inc., San Diego, CA | 1:50 for FACS |
| CD133 | Cell Signaling Technology, Beverly, MA | 1:100 for IHC |
| F4/80 | Proteintech Group, China | 1:100 for IHC |

**Table S10. Primer list.**

| **Gene** | **Forward primer (5’-3’)** | **Reverse primer (5’-3’)** |
| --- | --- | --- |
| MCB1(Human) | Forward (5*′*- 3*′*) | GTGACTTTGAATTTGGAGTAG |
|  | Reverse (5*′*- 3*′*) | ATCGTCTGAGTCTTCAGTCC |
| p53(Human) | Forward (5*′*- 3*′*) | ACCTATGGAAACTACTTCCTGAAA |
|  | Reverse (5*′*- 3*′*) | CTGGCATTCTGGGAGCTTCA |
| EpCAM(Human) | Forward (5*′*- 3*′*) | CGCAGCTCAGGAAGAATGTG |
|  | Reverse (5*′*- 3*′*) | TGAAGTACACTGGCATTGACGA |
| CD24(Human) | Forward (5*′*- 3*′*) | GCAAACAGATGTGTTCTTAAT |
|  | Reverse (5*′*- 3*′*) | TCATCCCTAAGATCAAGTTT |
| CD133(Human) | Forward (5*′*- 3*′*) | AGAGGAAGCCGCAAC |
|  | Reverse (5*′*- 3*′*) | CTGGCTCGTGAATTATTTAT |
| CD90(Human) | Forward (5*′*- 3*′*) | GAATACGGAAATGGATTAAG |
|  | Reverse (5*′*- 3*′*) | GTATTCATTTCCTCTGGTCT |
| SOX2(Human) | Forward (5*′*- 3*′*) | TGGAGAAGGAATGGTCCACTTC |
|  | Reverse (5*′*- 3*′*) | GGATAAGTACACGCTGCCCG |
| OCT4(Human) | Forward (5*′*- 3*′*) | ATGTGCGCGTAACTGTCCAT |
|  | Reverse (5*′*- 3*′*) | CTGCAGTGTGGGTTTCGGGCA |
| Nanog (Human) | Forward (5*′*- 3*′*) | AATACCTCAGCCTCCAGCAGATG |
|  | Reverse (5*′*- 3*′*) | TGCGTCACACCATTGCTATTCTTC |
| c-Myc(Human) | Forward (5*′*- 3*′*) | CCCTCCACTCGGAAGGACTA |
|  | Reverse (5*′*- 3*′*) | GCTGGTGCATTTTCGGTTGT |
| β-actin (Human) | Forward (5*′*- 3*′*) | GGCCCAGAATGCAGTTCGCCTT |
|  | Reverse (5*′*- 3*′*) | AATGGCACCCTGCTCACGCA |
| MCB1-FRT (Mouse) | Forward (5*′*- 3*′*) | CTACCCTGTGAAACGGTCATCCAAC |
|  | Reverse (5*′*- 3*′*) | GCAGGCTGTCTGTGGACTCACATC |
| MCB1-P1 (Mouse) | Forward (5*′*- 3*′*) | CCGGCCGGGCCTCGTCGTCT |
| MCB1-P2 (Mouse) | Forward (5*′*- 3*′*) | GAGAATAGGCCCAAATGTGGAACAC |
| MCB1-P3 (Mouse) | Forward (5*′*- 3*′*) | GGGACAGGATAAGTATGACATCATC |
| Alb-Cre (Mouse) | Forward (5*′*- 3*′*) | ATGCCCAAGAAGAAGAGGAAGGT |
|  | Reverse (5*′*- 3*′*) | GAAATCAGTGCGTTCGAACGCTAGA |
| FGFR1 (Human) | Forward (5*′*- 3*′*) | ATTTCTGCCTTGGCCCTACC |
|  | Reverse (5*′*- 3*′*) | CTAGCGCAGTCTTTGGGGAA |
| VEGFR3 (Human) | Forward (5*′*- 3*′*) | GGCCGCCAGGTATTACAACT |
|  | Reverse (5*′*- 3*′*) | TGTCTGGTTGTCCACAGAGC |
| si-MCB1 | 5’- GCAGGAUGCUGUCAACAUATT -3’ | |
| si-p53 | 5’- GTACCACCATCCACTACAA -3’ | |
| si-FGFR1 | 5’-GCCAAGACAGTGAAGTTCAAA-3’ | |
| si-VEGFR3 | 5’-GAAGCCCAAUCAAUAACUGUU -3’ | |
